# Linking Shifts in Bacterial Community Composition and Function with Changes in the Dissolved Organic Matter Pool in Ice-Covered Baiyangdian Lake, Northern China

**DOI:** 10.3390/microorganisms8060883

**Published:** 2020-06-11

**Authors:** Shilei Zhou, Yue Sun, Minghui Yu, Zhenpeng Shi, Hang Zhang, Ruizhe Peng, Zaixing Li, Jiansheng Cui, Xiao Luo

**Affiliations:** Pollution Prevention Biotechnology Laboratory of Hebei Province, School of Environmental Science and Engineering, Hebei University of Science and Technology, Shijiazhuang 050018, China; hbkjdxsy@126.com (Y.S.); yuyjjkxf@126.com (M.Y.); hbkjdxszp@126.com (Z.S.); zhfjdxsy@163.com (H.Z.); przwydszh@163.com (R.P.); li_zaixing@163.com (Z.L.); cui1603@163.com (J.C.); luoxnk@126.com (X.L.)

**Keywords:** chromophoric dissolved organic matter (CDOM), excitation-emission matrix-parallel factor analysis (EEM-PARAFAC), bacterial community, functional composition, Network analysis, Baiyangdian Lake

## Abstract

The relationship between CDOM (Chromophoric dissolved organic matter) and the bacterial community was investigated in ice-covered Baiyangdian Lake. The results showed that environmental parameters significantly differed in Baiyangdian Lake, whereas *a*-diversity was not significantly different. Moreover, the microbial and functional communities exhibited significant differences, and T (Temperature), pH, ORP (Oxidation-reduction potential), DO (Dissolved oxygen), NO_3_^−^-N, NH_4_^+^-N, and Mn (Manganese) were the main environmental factors of these differences, based on redundancy analysis and the Mantel test. Biomarkers of the microbial and functional communities were investigated through linear discriminant analysis effect size and STAMP analysis. The number of biomarkers in the natural area was highest among the typical zones, and most top functions were related to carbohydrate metabolism. Two protein-like components (C1 and C2) and one humic-like component (C3) were identified by parallel factor analysis, and C1 was positively related to C2 (*R* = 0.99, *p* < 0.001), indicating the same sources. Moreover, CDOM significantly differed among the typical zones (*p* < 0.001). The high biological index, fluorescence index, β:α, and low humification index indicated a strong autochthonous component and aquatic bacterial origin, which was consistent with the results of UV-vis absorption spectroscopy. Network analysis revealed non-random co-occurrence patterns. The bacterial and functional communities interacted closely with CDOM. The dominant genera were CL500-29_marine_group, *Flavobacterium*, *Limnohabitans*, and *Candidatus_Aquirestis*. Random forest analysis showed that C1, C2, and C3 are important predictors of *α*- and *β*-diversity in the water bacterial community and its functional composition. This study provides insight into the interaction between bacterial communities and DOM (Dissolved organic matter) in ice-covered Baiyangdian Lake.

## 1. Introduction

Bacteria, acted as important components of ecosystems, playing a crucial roles in nutrient cycles [1]. Various pollutants are closely related to the differences in the bacterial community composition, abundance, and diversity in freshwater ecosystems. Dissolved organic matter (DOM), which acts as one of the largest organic carbon pools in the biosphere [2], a complex, heterogeneous, and polymorphous mixture in natural waters. Moreover, DOM contributes to bacterial metabolism and affects the availability of inorganic nutrients, as well as greatly influences the microbial community structure and functional composition. Furthermore, CDOM is an important component of DOM reflecting the characteristics of DOM in the environment.

In recent years, fluorescence measurements of CDOM, based on EEM-PARAFAC analysis, exhibited further advantages in characterizing the spectral properties and sources of CDOM [3]. The effectiveness of this technique in water quality analysis has been demonstrated in studies of lakes [4], estuaries [5], rivers [6], and reservoirs [7,8]. Most studies have focused on the variations and environmental driving factors of bacterial communities in lakes [9,10]. However, the relationships between the microbial community composition and characteristics of CDOM in lakes remain poorly understood. Few studies have reported on the intrinsic relationships between bacterial communities and CDOM components. For example, the linkages between the bacterial community composition and CDOM were investigated in Lake Taihu ecosystems [2] and the CDOM composition and relationship between CDOM and the microbial community in Lake Chaohu and its inflow rivers were examined [4]. The interactions between the microbial community and DOM in freshwater ecosystems were explored in Lake Carioca [1]. The response to the various DOM of the composition and function of bacterial communities has been analysed at river confluences in urban areas [11]. Previous studies have mostly focused on southern lakes in China. However, the specific relationship between the composition of CDOM and bacterial community structure and functional composition in Baiyangdian Lake in northern China is unclear. Previous studies of Baiyangdian Lake mainly focused on water quality evolution [12], ecological risk assessment [13], aquatic macrophyte variation [14], antibiotic distribution [15], and pollutant release fluxes [16].

Moreover, Baiyangdian Lake is ice-covered from December to February, which has important effects on shifts in the bacterial community structure, functional composition, and carbon cycle. Aquatic bacterial communities play important roles in nutrient cycling in watershed ecosystems. However, few studies have examined the interaction between microbial and functional communities and CDOM in freshwater ecosystems. Therefore, in this study, we; (1) characterized the composition, source, and distribution of CDOM; (2) explored the characteristics of the bacterial community structure and functional composition; and (3) analysed the relationship between the components of CDOM and bacterial composition in ice-covered Baiyangdian Lake.

## 2. Materials and Methods

### 2.1. Research Area and Sample Collection

Baiyangdian Lake (37°45′–39°00′N, 115°45′–116°03′E), located in Xiong′an New Area, exhibited a total area of 366 km^2^ and average water depth of approximately 3 m. Baiyangdian provides important ecological opportunities for economic development in the surrounding areas. Particularly, after establishment of the Xiong′an New Area, Baiyangdian, Bohai, and Danjiangkou were defined as the “New Three Lakes,” and the water quality and ecological status of the water bodies became important factors in the construction of Xiong′an New Area. Additionally, the structure of Baiyangdian Lake is unique; the water body is shallow and fluctuating and is divided into a natural area (N.A), tourist area (B.A), living area (L.A), breeding area (B.A), and estuary area (E.A). Baiyangdian Lake has 6 major inflow rivers, including the Ping River (PH), Bao River (BH), Fu River (FH), Baigouyin river (BGYH), Zhulong River (ZLH), and Tang River (TH).

**Figure 1:**
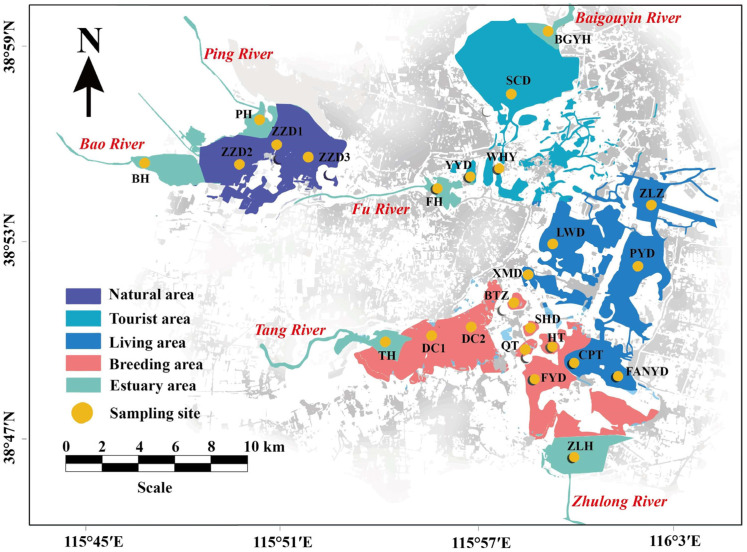
Location of sampling sites in Baiyangdian Lake, Xiongan New Area of China.

In this study, 25 water samples were collected from 5 typical zones in Baiyangdian Lake (Figure 1). The natural area contained 3 sample sites (ZZD1, ZZD2, and ZZD3); the tourist area included 3 sample sites (WHY, YYD, and SCD); living area contained 6 sample sites (ZLZ, PYD, FANYD, CPT, XMD, and LWD); breeding area contained 7 sample sites (FYD, HT, QT, SHD, BTZ, DC1, and DC2); and estuary area included 6 major inflow rivers (PH, BH, FH, BGYH, ZLH, and TH) with industrial, domestic, and agricultural pollution.

Water sampling was performed on January 15, 2019 by collecting 5 L water in the medium water layer in every water sample site. The samples were transported to the laboratory, and all parameters (water parameters and fluorescence) were measured within 48 h. Next, 2 L water sample was filtered through a 0.22-μm cellulose acetate filter membrane and stored at −80 °C until DNA extraction.

### 2.2. Measurement of Environmental Parameters

A multi-parameter water quality analyser (Hydrolab DS5, Loveland, CO, USA) was used to measure the water temperature (T), dissolved oxygen (DO), pH, oxidation-reduction potential (ORP), electrical conductivity (EC), and chlorophyll-*a* (CHl-*a*) at each sample site. In the laboratory, chemical analyses [nitrate (NO_3_^−^-N), nitrite (NO_2_^−^-N), ammonia (NH_4_^+^-N), total nitrogen (TN), total dissolved nitrogen (TDN), total phosphorus (TP), total dissolved phosphorus (TDP), permanganate index (COD_Mn_), Fe, and Mn] of the water samples were performed using standard methods [17].

### 2.3. Spectral Characteristics of CDOM

Detail information about measurements of EEM spectroscopy (F-7000, Hitachi, Japan) and UV–visible absorption spectroscopy (DR6000, Ames, IA, USA), fluorescence spectroscopy analyses, and UV–visible absorption spectroscopy analyses could be found in the Supplementary Materials. Moreover, the spectral characteristics of CDOM were investigated through the following indices of CDOM, these indices included fluorescence index (FI) [7], biological index (BIX) [18], humification index (HIX) [19], freshness index (β:α) [7], *F*n280 [20], *F*n355 [20], E2/E3 [21], E3/E4 [21], E4/E6 [22], and *S*_R_ value [23], respectively.

### 2.4. DNA Extraction and PCR Amplification

DNA was extracted using a Water DNA Kit (OMEGA, Irving, TX, USA) and then purified. Universal primers 338F and 806R were used for PCR amplification according to standard protocols at Shanghai Majorbio Bio-pharm Technology Co., Ltd. (Shanghai, China). The PCR mix and reaction protocol were consistent with those in our previous research [7]. Moreover, the sequencing data was deposited in the National Center for Biotechnology Information (NCBI, https://submit.ncbi.nlm.nih.gov/subs/sra/) database with the accession number PRJNA636304.

### 2.5. Sequence Analysis

#### 2.5.1. Water Bacterial Community Diversity

In this study, the OTUs representing < 0.1% of the total 16S rRNA reads have be eliminated from analysis. α-Diversity indices, including community richness estimators (Chao1 and ACE) and community diversity indices (Shannon index, Simpson index, and coverage), were determined using R.3.5.3 (vegan package) [24]. The β-diversity indices of the water microbial community were used to explore differences in the microbial communities through principal co-ordinates analysis (PCoA) using R.3.5.3 (vegan package). Linear discriminant analysis with (LDA = 3) was performed to investigate the significantly different biomarkers [25]. Analysis of similarities (ANOSIM), permutation multivariate analysis of variance (Adonis), and multi-response permutation procedure (MRPP) were used to analyse the spatial differences in the water microbial community structure through R.3.5.3 (vegan package). The functional compositions of bacterial were generated using PICRUSt [26], Tax4Fun [27], and FAPROTAX [28].

#### 2.5.2. Key Environment Factor Analysis

Redundancy analysis (RDA) was used to evaluate the relationship between the bacterial community structure and water environmental parameters with variance inflation factor analysis (VIF < 10) [29,30]. The mantel test was performed to investigate the relationships between water microbial/functional communities and environmental parameters [31]. Random forest (RF) analysis was used to explore the main driving factors of the α- and β-diversity of the water bacterial community and its functional composition using the “rfPermute” package [32].

#### 2.5.3. Network Analysis

Network analysis was performed to explore the interactions between the microbial populations and CDOM components. Topological features included the total node number, total edge number, clustering coefficient, network diameter, average path length, closeness centrality, network density, betweeness centrality, degree centralization, total module number, and modularity. Based on the generalized random graph model technique, a stochastic network with the most uniform number of nodes and edges compared to those in the real network was created. Parameters of random networks were generated from 1000 randomly rewired networks. Network analysis was performed using R.3.5.3 (psych, igraph and vegan packages). The networks were visualized using Gephi software (0.9.2).

## 3. Results and Discussion

### 3.1. Spatial Differences in Environmental Parameters

**Figure 2:**
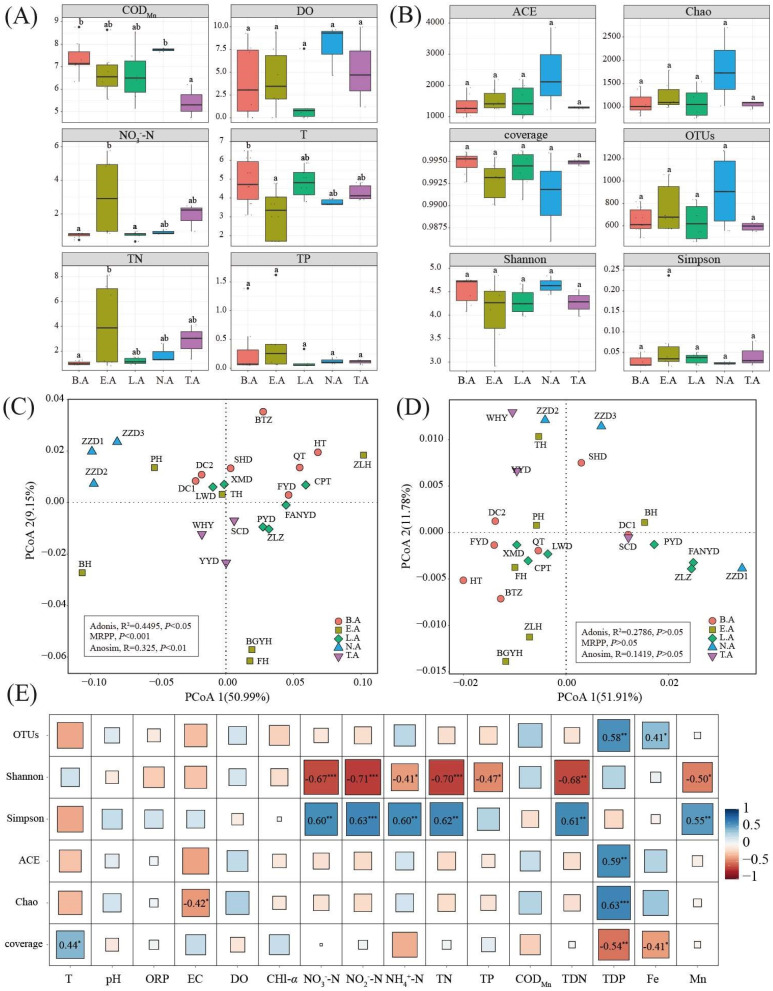
Alpha diversity and environment factors in Baiyangdian Lake. (**A**) environment factors (different letters are significantly different (*p* < 0.05)); (**B**), Alpha diversity (different letters are significantly different (*p* < 0.05)); (**C**), PCoA of alpha diversity; (**D**), PCoA of environment factors; (**E**) correlation between environment factors and alpha diversity (*, **, *** indicate the significance of the correlation at *p* < 0.05, *p* < 0.01, and *p* < 0.001).

A wide range of environmental conditions was investigated in ice-covered Baiyangdian Lake (Figure 2A). Particularly, the water temperature ranged from 1.69 °C to 6.51 °C; the water temperature of E.A exhibited a minimum value of 3.15 ± 1.26 °C and significantly differed from that of B.A. The DO concentration did not significantly differ among the typical zones in Baiyangdian Lake, with L.A exhibiting the lowest value of 1.69 ± 2.92 mg/L and N.A showing the highest value of 5.65 ± 3.66 mg/L. The COD_Mn_ of T.A reached a minimum value of 4.90 ± 2.59 mg/L, which significantly differed from those of B.A and N.A. The maximum value of NO_3_^−^-N was observed in E.A (FH, 5.76 mg/L), and the concentration of NO_3_^−^-N for E.A reached 3.06 ± 2.35 mg/L, significantly differing from those in B.A and L.A. TN showed a similar trend as NO_3_^−^-N, ranging from 0.84 to 8.13 mg/L. TP exhibited no significant difference among the typical zones in Baiyangdian Lake. PCoA demonstrated that PCoA1 and PCoA2 accounted for 50.99% and 9.15% of variation, respectively. The sample sites in the same zone showed tighter clustering (except for E.A), whereas those from different zones were relatively widely distributed. Furthermore, environmental parameters significantly differed among the typical zones in Baiyangdian Lake (Adonis, *p* < 0.05; MRPP, *p* < 0.001; Anosim analysis, *p* < 0.01).

### 3.2. Alpha Diversity of Bacterial Communities

Clustering and comparison revealed 17,346 operational taxonomic units (OTUs) in all samples based on a 0.97 threshold. The measures of ACE index, Chao richness index, coverage value, OTUs, Shannon diversity index, and Simpson diversity index did not significantly differ (*p* > 0.05) among the typical zones in Baiyangdian Lake. In detail, the ACE index of ZZD1 (N.A) exhibited the highest value of 3854.1, whereas that of XMD (L.A) exhibited the lowest value of 944.68. The Chao richness index exhibited a similar trend, ranging from 759.4 (L.A, XMD) to 2701.1 (N.A, ZZD1). The coverage values of all samples were > 0.98, indicating that the sequencing depth was enough to reflect real bacterial information. The number of OTUs ranged from 460 (L.A, XMD) to 1272 (N.A, ZZD1) in Baiyangdian Lake. The Shannon index and Simpson index ranged from 2.91 (E.A, BGYH) and 0.02 (E.A, PH) to 4.85 (N.A, ZZD1) and 0.24 (E.A, BGYH) in Baiyangdian Lake. PCoA showed that the PCoA1 and PCoA2 together accounted for 63.69% of variation, with no significant difference among the typical zones in Baiyangdian Lake (Adonis, *p* > 0.05; MRPP, *p* > 0.05; Anosim analysis, *p* > 0.05). The correlation between diversity and environmental parameters was also investigated. In detail, the Shannon index was negatively related to NO_3_^−^-N (R = −0.67, *p* < 0.001), NO_2_^−^-N (R = −0.71, *p* < 0.001), NH_4_^+^-N (R = −0.41, *p* < 0.05), TN (R = −0.70, *p* < 0.001), TP (R = −0.47, *p* < 0.05), TDN (R = −0.68, *p* < 0.05), and Mn (R = −0.50, *p* < 0.05). The Simpson index was positively related to NO_3_^−^-N (R = 0.60, *p* < 0.01), NO_2_^−^-N (R = 0.63, *p* < 0.001), NH_4_^+^-N (R = 0.60, *p* < 0.01), TN (R = 0.62, *p* < 0.01), TDN (R = 0.61, *p* < 0.01), and Mn (R = 0.55, *p* < 0.01). Total dissolved phosphorus exhibited a positive correlation with OTUs (R = 0.58, *p* < 0.01), ACE index (R = 0.59, *p* < 0.01), Chao (R = 0.63, *p* < 0.001) and negatively correlation with coverage (R = −0.54, *p* < 0.01).

### 3.3. Spatial Distribution of Bacterial Communities

In response to the DOM loads, the individual populations (OTU >1%) were examined and found to contribute significantly to differences in the community composition at the phylum, class, and genus levels in Baiyangdian Lake (Figure S1). At the phylum level (Figure S1A), the ten dominant phyla were Proteobacteria (25.80–73.09%), Actinobacteria (2.51–40.89%), Bacteroidetes (3.36–32.44%), Cyanobacteria (1.47–34.46%), Verrucomicrobia (0.06–11.26%), Firmicutes (0.10–7.24%), Planctomycetes (0.01–4.07%), Epsilonbacteraeota (0.00–10.86%), Deinococcus-Thermus (0.05–1.41%), and Patescibacteria (0.19–1.29%). These dominant phyla exhibited significant differences (*p* < 0.001) among the typical zones in Baiyangdian Lake. In detail, Proteobacteria of E.A (32.15–72.73%) accounted for 48.31 ± 19.54% of the bacterial community and was higher than B.A (30.83 ± 1.76%), T.A (26.20 ± 14.37%), L.A (33.53 ± 6.82%), and N.A (35.26 ± 17.16%). The relative abundance ranks based on Actinobacteria were as follows: B.A (27.56 ± 7.45%), L.A (26.83 ± 10.96%), T.A (16.01 ± 7.44%), N.A (15.66 ± 6.55%), and E.A (14.27 ± 10.76%). The relative abundance ranks based on Bacteroidetes were B.A (20.35 ± 4.83%), E.A (19.84 ± 10.67%), L.A (17.21 ± 2.66%), T.A (16.32 ± 6.97%), and N.A (14.34 ± 6.95%). The relative abundance ranks based on Cyanobacteria were T.A (20.18 ± 10.54%), N.A (16.04 ± 6.93%), L.A (15.48 ± 11.33%), B.A (13.61 ± 7.19%), and E.A (9.16 ± 5.40%). These phyla are known to function in the carbon cycle in the water environment [33]. For instance, Proteobacteria play an important role in carbon metabolism [34], decompose soluble sugars [35] and can be influenced by humic-like DOM [36]. Actinobacteria play important roles in decomposing organic carbon for lake water quality [37,38] and were reported to be strongly affected by humic-like DOM [36]; Bacteroidetes are mainly responsible for carbon metabolism, including decomposing hydrolytic cellulose [39] and remineralising complex and labile DOM [40]. Cyanobacteria typically act as the dominant primary producers of DOM [41]. Planctomyces can remove organic matter pollutants [42].

At the class level (Figure S1B), the distribution of class (OTU >1%) was determined. The dominant classes included Gammaproteobacteria (16.09–64.89%), Actinobacteria (2.51–40.89%), Bacteroidia (3.36–32.40%), Oxyphotobacteria (1.47–34.46%), Alphaproteobacteria (3.58–29.12%), Verrucomicrobiae (0.06–11.26%), Bacilli (0.06–7.11%), Deltaproteobacteria (0.10–2.27%), Planctomycetacia (0.01–3.36%), Campylobacteria (0.00–10.86%), Deinococci (0.02–1.76%), Phycisphaerae (0.00–1.72%), and Saccharimonadia (0.01–1.20%). These classes exhibited significant differences in Baiyangdian Lake. In detail, Gammaproteobacteria in E.A were the most abundant at 35.88 ± 16.26% which was higher than the levels in B.A (20.88 ± 1.79%), T.A (18.56 ± 2.90%), L.A (22.13 ± 3.60%), and N.A (25.09 ± 3.80%). The relative abundance ranks based on Actinobacteria were as follows: L.A (26.83 ± 10.96%), B.A (26.22 ± 7.67%), T.A (15.01 ± 2.39%), E.A (14.27 ± 10.76%), and N. A (13.50 ± 2.33%). The relative abundance ranks based on Bacteroidia were as follows: B.A (22.06 ± 6.35%), E.A (19.81 ± 10.64%), T.A (18.81 ± 3.72%), L.A (17.21 ± 2.66%), and N.A (17.20 ± 3.42%). The relative abundance ranks based on Oxyphotobacteria were as follows: T.A (26.69 ± 7.24%), N.A (17.78 ± 8.94%), L.A (15.47 ± 11.32%), B.A (12.46 ± 7.24%), and E.A (9.13±5.37%). The relative abundance ranks based on Alphaproteobacteria were as follows: N.A (18.68 ± 2.55%), T.A (13.78 ± 3.95%), E.A (11.73 ± 8.90%), L.A (11.05 ± 4.10%), and B.A (10.55 ± 2.43%). Gammaproteobacteria can degrade organic matter [43]. Alphaproteobacteria and Actinobacteria have been reported to decompose humic-rich substances [44].

**Figure 3:**
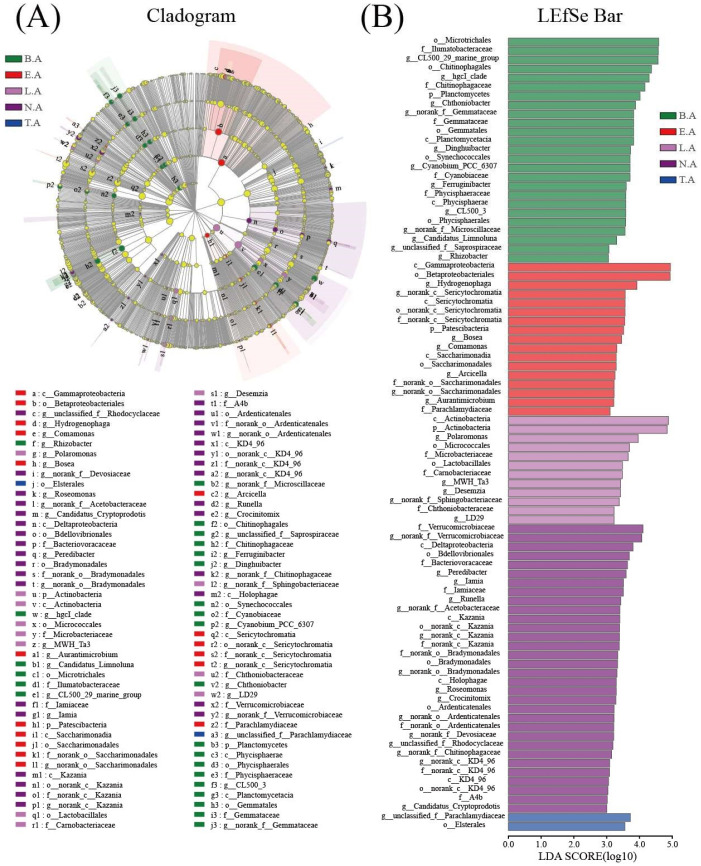
LEfSe analysis of microbial community in Baiyangdian Lake. (**A**) Cladogram; (**B**) LEfSe Bar.

At the gene level, the variations in the top 50 genera are shown in Figure S1C. The dominant genus were *Rhodoferax* (2.00–54.46%), norank_o__*Chloroplast* (0.93–34.40%), *Flavobacterium* (1.85–21.90%), CL500-29_marine_group (0.02–19.72%), unclassified_f__*Rhodobacteraceae* (1.08–13.75%), norank_f__*Sporichthyaceae* (0.60–11.86%), hgcI_clade (0.45–9.64%), Limnohabitans (0.91–8.82%), *Hydrogenophaga* (0.01–8.24%), *Luteolibacter* (0.03–8.22%), *Acinetobacter* (0.01–7.27%), *Sphingorhabdus* (0.36–7.20%), *Candidatus_Aquirestis* (0.11–6.10%), and *Polaromonas* (0.52–5.39%). Most of the dominant genera were related to carbon metabolism. *Flavobacterium* may promote the degradation of organic substances and removal of nitrogen and phosphorus [45,46]. CL500-29 bacteria are considered as generalists and can utilize a variety of DOC under aerobic conditions [47]. *Rhodobacteraceae* can utilize organic matter to grow [48]. Acinetobacter can aerobically decompose large organic molecules [49]. Limnohabitans and *Candidatus_Aquirestis* consume LMW carbon [50]. *Cytophaga* and *Fibrobacter* can degrade cellulolysis [51,52]. *Verrucomicrobiaceae* is involved in degrading complex microbial-produced DOM [1]. LEfSe was performed to evaluate the microbial community, which significantly differed among the typical zones in Baiyangdian Lake. LDA scores higher than 3 were used to identify bacterial groups with significant differences (Figure 3). Twenty-five major genera were enriched in B.A: *Microtrichales*, *Ilumatobacteraceae*, CL500_29_marine_group, *Chitinophagales*, *hgcI*_*clade*, *Chitinophagaceae*, *Planctomycetes*, *Chthoniobacter*, *Gemmataceae*, *Gemmatales*, *Dinghuibacter*, *Synechococcales*, *Cyanobium*_PCC_6307, *Cyanobiaceae*, *Ferruginibacter*, *Phycisphaeraceae*, *Phycisphaerae*, CL500_3, *Phycisphaerales*, *Microscillaceae*, *Candidatus*_*Limnoluna*, *Saprospiraceae*, and *Rhizobacter*. The 17 genera enriched in E.A were *Gammaproteobacteria*, *Betaproteobacteria*, *Hydrogenophaga*, *Sericytochromatia*, *Patescibacteria*, *Bosea*, *Comamonas*, *Saccharimonadia*, *Saccharimonadales*, *Arcicella*, *Aurantimicrobium*, and *Parachlamydiaceae*. Twelve genera were enriched in L.A, namely, *Actinobacteria*, *Polaromonas*, *Micrococcales*, *Microbacteriaceae*, *Lactobacillales*, *Carnobacteriaceae*, *MWH_Ta3*, *Desemzia*, *Sphingobacteriaceae*, *Chthoniobacteraceae*, and LD29. Thirty-two major genera were enriched in N.A., namely, *Verrucomicrobiaceae*, *Deltaproteobacteria*, *Bdellovibrionales*, *Bacteriovoracaceae*, *Peredibacter*, *Iamia*, *Iamiaceae*, *Runella*, *Acetobacteraceae*, *Kazania*, *Bradymonadales*, *Holophagae*, *Roseomonas*, *Crocinitomix*, *Ardenticatenales*, *Devosiaceae*, *Rhodocyclaceae*, *Chitinophagaceae*, KD4_96, A4b, *Candidatus*_*Cryptoprodotis*, *Parachlamydiaceae*, and *Elsterales*. *Parachlamydiaceae* and *Elsterales* were enriched in T.A. N.A showed the largest number of biomarkers highest among the typical zones in Baiyangdian Lake. Furthermore, in order to investigate the variations of microbial structure, the PCoA analysis based on OTUs was carried out and the results of PCoA revealed that the first two principal factors (PCoA1 and PCoA2) explained 23.67% and 15.34% of the total variance, respectively (Figure S2). Meanwhile, the microbial structure exhibited significant difference based on Adonis (*p* < 0.01), MRPP (*p* < 0.001), and ANOSIM (*p* < 0.01), respectively.

### 3.4. Comparison of Functional Properties

Based on PICRUSt2 (level-2), Tax4Fun (level-2), and FARPROTAX, bacterial community functions were predicted in Baiyangdian Lake (Figure S2). Based on PICRUSt2 (Figure S2A), the dominant functions were carbohydrate metabolism, global and overview, amino acid metabolism, energy metabolism (related to carbon fixation), metabolism of cofactors and vitamins, membrane transport, nucleotide metabolism, translation, replication and repair, cellular community-prokaryotes, and lipid metabolism. Based on Tax4Fun (Figure S2B), the dominant functions were carbohydrate metabolism, amino acid metabolism, membrane transport, energy metabolism, metabolism of cofactors and vitamins, and signal transduction. Based on FARPROTAX (Figure S2C), the dominant functions were chemoheterotrophy, aerobic_chemoheterotrophy, chloroplasts, fermentation, photoheterotrophy, phototrophy, and methanotrophy. Most top functions were related to carbohydrate metabolism. Previous studies showed that both amino acid metabolism and cofactor and vitamin metabolism are strongly associated with the degradation of alanine, aspartate, glutamate, and other carbohydrates [53].

**Figure 4:**
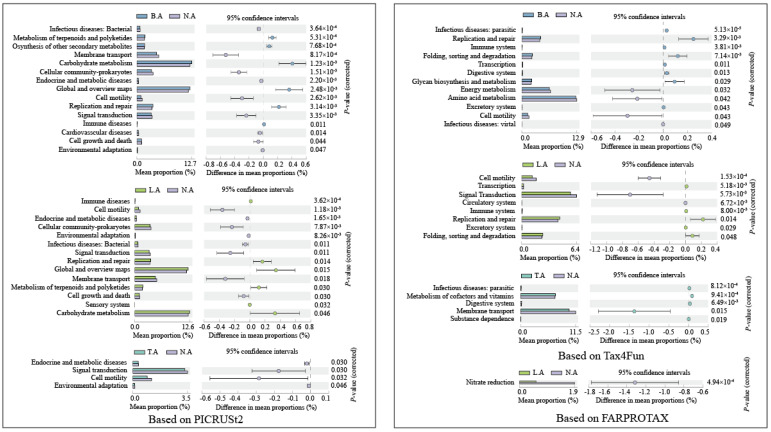
The functional composition of significant changes based on PICRUSt2, Tax4Fun, and FARPROTAX using the response ratio method at a 95% confidence interval (CI).

These major gene categories showed significantly different abundances among the microbial communities in different typical zones in Baiyangdian Lake (*p* < 0.05, Figure 4). Based on PICRUSt2, Compared to N.A, the functional genes involved in carbohydrate metabolism and global and overview (carbon metabolism) were significantly higher in B.A and L.A (*p* < 0.05). This indicates that biodegradation of labile substances (e.g., amino acids) was less active and resulted in higher percentages of humic components in N.A. Based on Tax4Fun, compared to N.A, the functional genes linked to amino acid metabolism (B.A), signal transduction (L.A), and membrane transport (T.A) were significantly lower (*p* < 0.05), whereas replication and repair (B.A and L.A) and metabolism of cofactors and vitamins (T.A) were significantly higher (*p* < 0.05). Compared to N.A, only nitrate reduction (L.A) was significantly lower (*p* < 0.05) based on FARPROTAX.

### 3.5. Key Environment Factor Analysis

**Figure 5:**
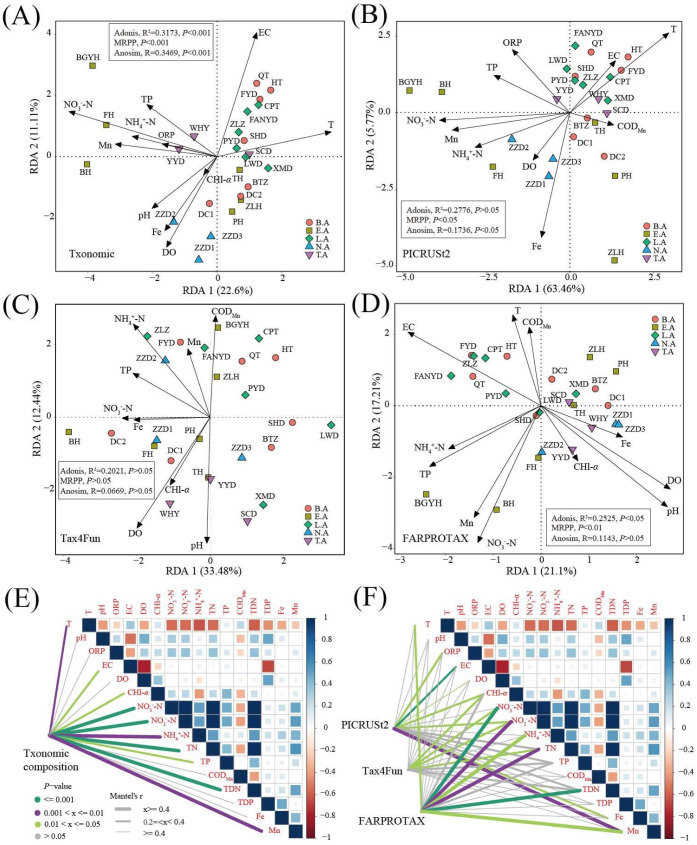
RDA, and Mantel test of taxonomic community and functional community in Baiyangdian Lake. (**A**) RDA of taxonomic community; (**B**) RDA of functional community based on PICRUSt2; (**C**) RDA of functional community based on Tax4Fun; (**D**) RDA of functional community based on FARPROTAX; (**E**) Mantel test between taxonomic community and environment factors; (**F**) Mantel test between functional community and environment factors.

RDA and the Mantel test were used to determine the relationship between environmental parameters and the microbial community (Figure 5). For the taxonomic composition, based on a VIF <10 (Table 1), the results of RDA (F = 2.156, *p* < 0.001) showed that RDA1 and RDA2 accounted for 22.6% and 11.11% of variation in the microbial community, and RDA1 played a main role in the distribution of microbial composition (Figure 5A). T, pH, ORP, NO_3_^−^-N, NH_4_^+^-N, TP, and Mn were key environmental factors in the microbial community. The microbial communities exhibited significant differences among the typical zones in Baiyangdian Lake (Adonis, *p* < 0.001; MRPP, *p* < 0.001; Anosim analysis, *p* < 0.001). Moreover, the results of the Mantel test showed that T (*R* = 0.38, *p* < 0.01), EC (*R* = 0.21, *p* < 0.05), CHl-α (*R* = 0.37, *p* < 0.05), NO_3_^−^-N (*R* = 0.70, *p* < 0.001), NO_2_^−^-N (*R* = 0.74, *p* < 0.001), NH_4_^+^-N (*R* = 0.50, *p* < 0.01), TN (*R* = 0.71, *p* < 0.001), TP (*R* = 0.33, *p* < 0.05), TDN (*R* = 0.70, *p* < 0.001), and Mn (*R* = 0.43, *p* < 0.01) were important environment factors (Figure 5E). For the functional composition (PICRUSt2), RDA1 and RDA2 together accounted for 69.23% of variation in the microbial community (F = 3.798, *p* < 0.001). T, ORP, EC, DO, NO_3_^−^-N, NH_4_^+^-N, TP, COD_Mn_, Fe, and Mn were key environmental factors in the microbial community based on VIF < 10 (Figure 5B). The functional community significantly differed among the typical zones in Baiyangdian Lake (Adonis, *p* > 0.05; MRPP, *p* < 0.05; Anosim, *p* < 0.05). The results of the Mantel test showed that ORP (*R* = 0.38, *p* < 0.05), EC (*R* = 0.33, *p* < 0.001), DO (*R* = 0.17, *p* < 0.05), CHl-*α* (*R* = 0.33, *p* < 0.05), NO_2_^−^-N (*R* = 0.28, *p* < 0.05), Fe (*R* = 0.36, *p* < 0.05), and Mn (*R* = 0.42, *p* < 0.01) were important environmental factors (Figure 5F). For the functional composition (Tax4Fun), RDA1 and RDA2 together accounted for 69.23% of variation in the microbial community (F = 1.922, *p* < 0.05). pH, DO, CHl-α, NO_3_^−^-N, NH_4_^+^-N, TP, COD_Mn_, Fe, and Mn were key environmental factors in the microbial community based on a VIF <10 (Figure 5C). The functional community did not significantly differ among the typical zones in Baiyangdian Lake (Adonis, *p* > 0.05; MRPP, *p* > 0.05; Anosim analysis, *p* > 0.05). The results of the Mantel test showed that CHl-*α* (*R* = 0.32, *p* < 0.05), NO_3_^−^-N (*R* = 0.30, *p* < 0.05), NO_2_^−^-N (*R* = 0.33, *p* < 0.05), and TN (*R* = 0.27, *p* < 0.05) were important environmental factors (Figure 5F). For the functional composition (FARPROTAX), RDA1 and RDA2 together accounted for 38.31% of variation in the microbial community (F = 1.533, *p* < 0.05). T, pH, EC, DO, CHl-α, NO_3_^−^-N, NH_4_^+^-N, TP, COD_Mn_, Fe, and Mn were key environmental factors in the microbial community based on a VIF <10 (Figure 5D). The functional community exhibited significant differences among the typical zones in Baiyangdian Lake (Adonis, *p* < 0.05; MRPP, *p* < 0.01). The results of Mantel test showed that T (*R* = 0.34, *p* < 0.05), NO_3_^−^-N (*R* = 0.65, *p* < 0.001), NO_2_^−^-N (*R* = 0.72, *p* < 0.01), NH_4_^+^-N (*R* = 0.53, *p* < 0.05), TN (*R* = 0.66, *p* < 0.01), TDN (*R* = 0.66, *p* < 0.001), and Mn (*R* = 0.40, *p* < 0.05) were important environment factors (Figure 5F).

### 3.6. CDOM Characteristics

#### 3.6.1. EEM Spectroscopy Analysis

**Figure 6:**
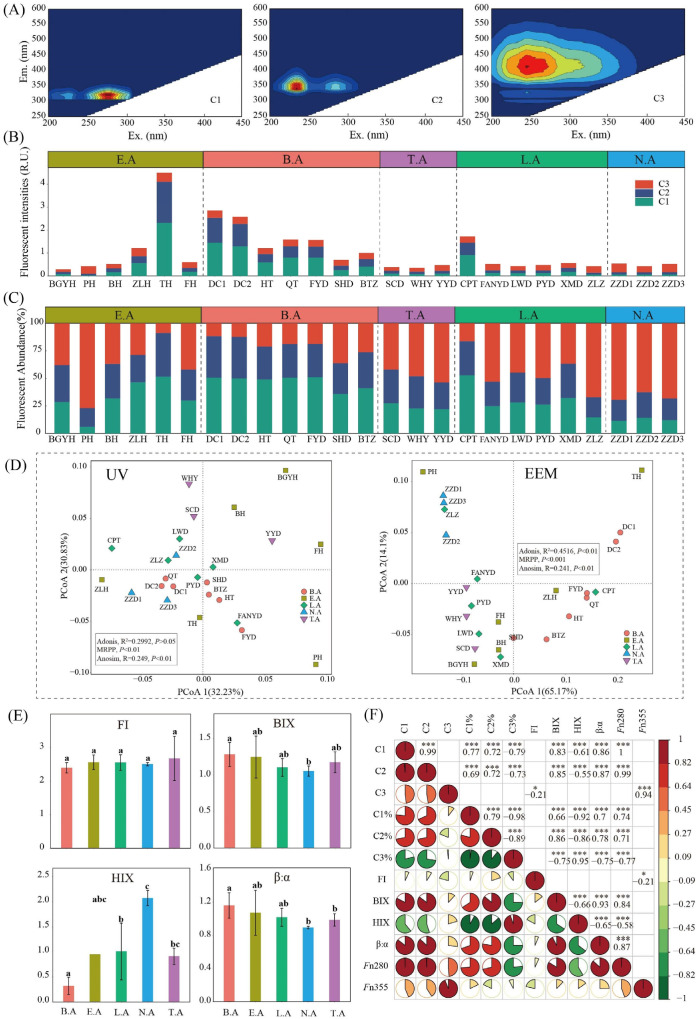
Fluorescence components and spatial distributions of fluorescence intensities and relative abundance in Baiyangdian Lake. (**A**) Fluorescence components; (**B**) Fluorescence intensities of fluorescence components; (**C**), Relative abundance of fluorescence components; (**D**) PCoA of CDOM based on UV and EEM; (**E**) Fluorescence indices; (**F**) correlation analysis (*, *** indicate the significance of the correlation at *p* < 0.05, and *p* < 0.001).

Comparisons of the EEM contours of each component with those reported previously (Table 2) showed that the three components identified from the fluorescence spectra consisted of two protein-like components and one humic-like component. Component 1 (C1: Ex/Em, 275/325 nm) was comparable to a protein-like substance [11] (Figure 6A). Component 2 (C2: Ex/Em, 225/345 nm) was considered to be similar to a protein-like substance (tryptophan-like DOM) [11] (Figure 6A). Component 3 (C3: Ex/Em, 250/410 nm) was likely related to a humic-like substance with a high molecular weight [54] (Figure 6A). The correlation between C1 and C2 reached 0.99 (*p* < 0.001), indicating that C1 and C2 had similar sources (Figure 6F). The total fluorescence intensities presented significant differences in Baiyangdian Lake (Figure 6B). Similar trends were observed for fluorescent components C1, C2, and C3. In detail, the fluorescent intensity of C1 ranged from 0.03 R.U. (PH) to 2.32 R.U. (TH), C2 ranged from 0.07 R.U. (PH) to 1.79 R.U. (TH), C3 ranged from 0.11 R.U. (BGYH) to 0.39 R.U. (TH), and total fluorescent intensity ranged from 0.29 R.U. (BGYH) to 4.50 R.U. (TH). The relative abundance of fluorescent intensity also significantly differed in Baiyangdian Lake (Figure 6C). In detail, the relative abundance of C1 ranged from 6.01% to 52.85%, C2 ranged from 17.09% to 39.81%, C3 ranged from 8.67% to 76.91%, and protein-like substances (C1+C2) ranged from 23.09% (PH) to 91.33% (TH), accounting for the main proportion of CDOM. Furthermore, we investigated the distributions of intensity and relative abundance of fluorescent components in the typical zones in Baiyangdian Lake. The fluorescent intensities of C1 and C2 exhibited similar trends; the ranks were B.A, E.A, L.A, T.A, and N.A (Figure S3B). The same ranks were observed based on the relative abundance of C1 and C2 (Figure S3B). The fluorescent intensity of C3 did not significantly differ among typical zones, whereas the relative abundance of C3 exhibited a significant difference (Figure S3B).

The distribution of CDOM based on EEM was investigated based on PCoA, with the results showing that PCoA1 and PCoA2 accounted for 65.17% and 14.1% of the variation in CDOM. Moreover, the CDOM exhibited significant differences among typical zones in Baiyangdian Lake (Adonis, *p* < 0.001; MRPP, *p* < 0.001; Anosim analysis, *p* < 0.001) (Figure 6D). FI is known to be correlated with DOM aromaticity and is often used as an indicator of DOM origin. The FI values in typical zones were >1.9 and exhibited no difference (*p* > 0.05), indicating low aromaticity and strong autochthonous component characteristics [55] (Figure 6E). The BIX values of typical zones were >1.0, indicating biological or aquatic bacterial origin [18] (Figure 6E). The HIX values of typical zones were <4.0, indicating strong autochthonous component characteristics [19] (Figure 6E). The ranks based on the freshness index (β:α) were B.A, E.A, L.A, T.A, N.A (Figure 6E), indicating that the proportion of newly generated DOM was decreased in this order. C1 and C2 were both significantly positively correlated with BIX, HIX, and *F*n280 (represented the relative abundance of protein-like substance), with the correlation coefficients reaching 0.83 (*p* < 0.001) and 0.85 (*p* < 0.001), 0.86 (*p* < 0.001) and 0.87 (*p* < 0.001), and 1.00 (*p* < 0.001) and 0.99 (*p* < 0.001), respectively (Figure 6F). Negatively relationships were observed with HIX (C1: *R* = −0.61, *p* < 0.001; C2: *R* = −0.55, *p* < 0.001) (Figure 6F). These results show that C1 and C2 belong to the low aromaticity and strong autochthonous component. C3 was positively related to *F*n355 (*R* = 0.94, *p* < 0.001) (represented by the relative abundance of humic-like substance), which was consistent with the results for the source (Figure 6F). C3 was positively related to COD_Mn_ (*R* = 0.52, *p* < 0.01) and Fe (*R* = 0.44, *p* < 0.05), whereas it was negatively related to NO_3_^−^-N (*R* = −0.45, *p* < 0.05) (Figure S3D). The detailed correlations are shown in Figure 6F and Figure S3D.

#### 3.6.2. UV–Visible Absorption Spectroscopy Analysis

The relative concentrations of CDOM were investigated using *α*_254_ and *α*_355_, respectively, (Figure S3A). The *α*_254_ and *α*_355_ exhibited similar trends, with ranks of N.A, B.A, E.A, L.A, and T.A (Figure S3A), which was consistent with the change in COD_Mn_. The *α*_254_ and *α*_355_ from T.A exhibited significant differences from B.A and L.A. The ranks based on E2/E3 were L.A, B.A, E.A, N.A, and T.A (Figure S3A), indicating that the molecular weight increased in this order in Baiyangdian Lake [21]. The E3/E4 values were all >3.5, indicating that the DOM had low humification characteristics [21]. The E4/E6 values did not significantly differ in Baiyangdian Lake. The *S*_R_ values showed that the molecular weight of T.A was highest among the typical zones in Baiyangdian Lake [23]. The results of PCoA showed that PCoA1 and PCoA2 together accounted for 63.06% and CODM (based on UV-vis) significantly differed among the typical zones in Baiyangdian Lake (MRPP, *p* < 0.01; Anosim analysis, *p* < 0.01) (Figure 6D). Moreover, NO_3_^−^-N and COD_Mn_ were dominant environmental factors affecting the CDOM based on UV-vis spectroscopy (Figure S3C). Specifically, NO_3_^−^-N was negatively related to E2/E4, A_254_/A_204_, *α*_254_, *α*_355_, *α*_440_, S_275-295_, S_350-400_, and *S*_R_ and positively correlated with A_220_/A_254_; COD_Mn_ was positively related to E2/E3, E2/E4, E2/E6, E3/E4, A_254_/A_204_, *α*_254_, *α*_355_, *α*_440_, S_275-295_, and S_350-400_ and positively correlated with A_220_/A_254_ and *S*_R_.

### 3.7. Co-Occurrence Network Analysis

**Figure 7:**
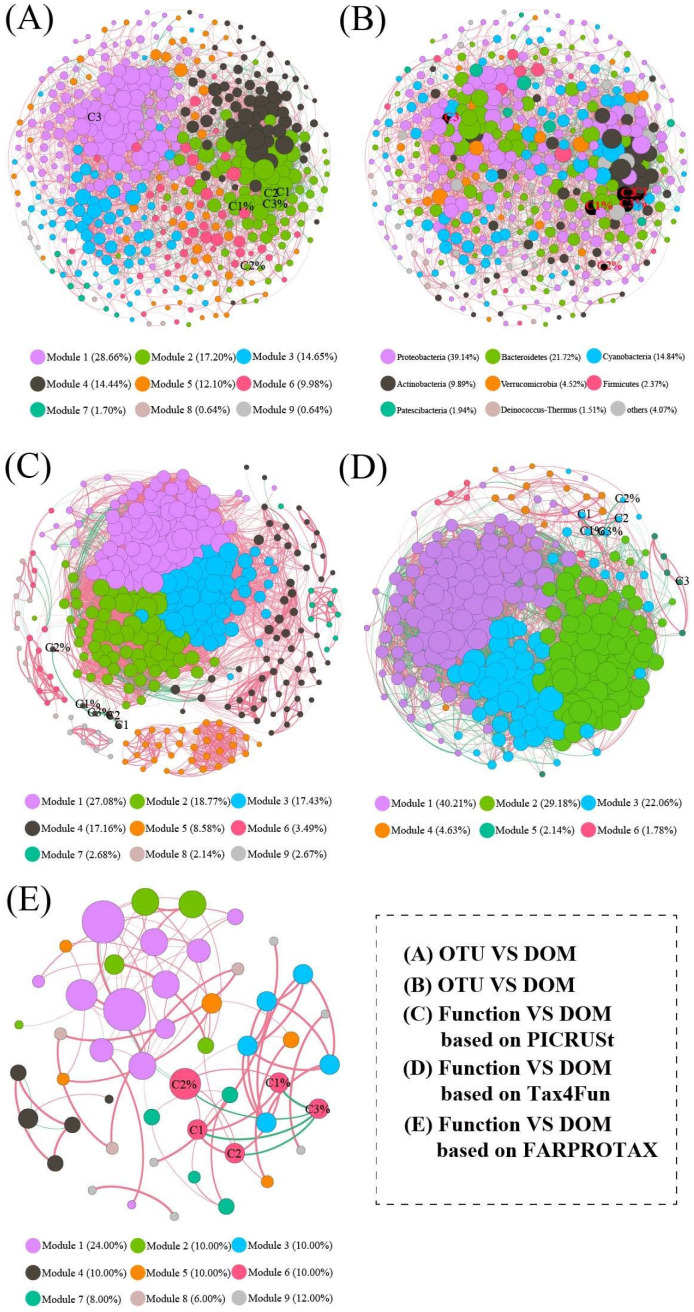
Network visualizes the OTU-DOM and functional community-DOM interactions in Baiyangdian Lake. Positive correlations were displayed in red and negative correlations were displayed in green. The nodes were coloured according to different types of modularity classes. The size of each node is proportional to the degree. (Spearman′s |r|> 0.6, *p* <0.05). (**A**) microbial network analysis based on OTU vs DOM (module level); (**B**) microbial network analysis based on OTU vs DOM (phylum level); (**C**) microbial network analysis based on function vs DOM (PICRUSt2); (**D**) microbial network analysis based on function vs DOM (Tax4Fun); (**E**) microbial network analysis based on function vs DOM (FARPROTAX).

Microbial community networks were used to investigate the interactions of OTUs based on the microbial composition (OTU level) and functional composition (PICRUSt2, Tax4Fun, and FARPROTAX) in Baiyangdian Lake (Figure 7). The values of modularity, average clustering coefficient, and average path length of the network were higher than in the random network (Table 3), suggesting that our network had “small-world” properties and a modular structure.

For the microbial composition, the OTU-OTU association network depicted in Figure 7A consists of 471 nodes and 3962 edges (Table 3). The network grouped microbial communities into 9 modules, accounting for 28.66% (module 1), 17.20% (module 2), 14.65% (module 3), 14.44% (module 4), 12.10% (module 5), 9.98% (module 6), 1.70% (module 7), 0.64% (module 8), and 0.64% (module 8). Network analysis showed that the positive edges accounted for 78.09%, indicating that symbiotic relationships accounted for most of the microbial network. The dominant phyla were Proteobacteria, Bacteroidetes, Cyanobacteria, Actinobacteria, Verrucomicrobia, Firmicutes, Patescibacteria, and Deinococus-Thermus, accounting for 39.14%, 21.72%, 14.84%, 9.89%, 4.52%, 2.37%, 1.94%, and 1.51%, respectively, (Figure 7B). Moreover, compared to the random network, the topological properties of the empirical networks were obviously higher than those of the random network (Table 3). Furthermore, C1, C2, C1%, C2%, and C3% belonged to module 2, whereas C3 belonged to module 1. In detail, 32 OTUs were related to C1, mainly belonging to module 2 (93.75%); these OTUs belonged to *CL500-29_marine_group*, *Flavobacterium*, *norank_Chloroplast*, *Luteolibacter*, *Candidatus Limnoluna*, and unclassified bacteria, respectively, (Table S1). Thirty-one OTUs were related to C1%, mainly belonged to module 2 (83.87%), and these OTUs belonged to *CL500-29_marine_group*, *Flavobacterium*, *norank_Chloroplast*, *Luteolibacter*, *hgcI_clade*, *Candidatus Limnoluna*, and unclassified bacteria, respectively (Table S2). Thirty-three 33 OTUs were related to C2, mainly belonging to module 2 (96.97%), and these OTUs belonged to *CL500-29_marine_group*, *Flavobacterium*, *norank_Chloroplast*, *Luteolibacter*, *Dinghuibacter*, and unclassified bacteria, respectively (Table S3). Five OTUs were related to C2%, mainly belonging to module 2 (60%), and these OTUs belonged to *Limnohabitans*, *Roseomonas*, *Sulfuricurvum*, *Luteolibacter*, and norank_o__*Gaiellales*, respectively (Table S4). Twenty-five OTUs were related to C3, mainly belonging to module 1 (100%), and these OTUs belonged to *CL500-29_marine_group*, *norank_f__T34*, *norank_Chloroplast*, *Flavobacterium*, *Rheinheimera*, and unclassified bacteria, respectively (Table S5). Twenty-six OTUs were related to C3%, mainly belonging to module 2 (92.31%). These OTUs belonged to *CL500-29_marine_group*, *Flavobacterium*, *norank_Chloroplast*, *Roseomonas*, *hgcI_clade*, *Candidatus Limnoluna*, and unclassified bacteria, respectively (Table S6). CL500-29 bacteria [47], *Flavobacterium* [45,46], *Limnohabitans* [50], and *Candidatus_Aquirestis* [50] have been reported to be related to carbon metabolism. Detailed information is shown in Tables S1–S6.

For functional composition based on PICRUSt2 (level 3), the network was divided into 9 modules by network partitioning, and the positive edges accounted for 99.08% (Figure 7C), with module 1 (27.08%), module 2 (18.77%), module 3 (17.43%), module 4 (17.16%), module 5 (8.58%), module 6 (3.49%), module 7 (2.68%), module 8 (2.14%), and module 9 (2.67%). Moreover, C1, C1, C2, C2, and C3 belonged to module 4 in this network. C1 was significantly related to mineral absorption (*R* = −0.64, *p* < 0.001) and the proteasome (*R* = 0.62, *p* < 0.001). C1% was positively related to mineral absorption (*R* = −0.67, *p* < 0.001) and the proteasome (*R* = 0.64, *p* < 0.001). C2 was significantly related to mineral absorption (*R* = −0.62, *p* < 0.001), the proteasome (*R* = 0.68, *p* < 0.001), and biosynthesis of type II polyketide products (*R* = 0.62, *p* < 0.001). C2% was positively related to alpha linolenic acid metabolism (*R* = 0.63, *p* < 0.001) and linoleic acid metabolism (*R* = 0.62, *p* < 0.01). C3% was related to mineral absorption (*R* = 0.63, *p* < 0.001) and the proteasome (*R* = −0.61, *p* < 0.01).

For functional composition based on Tax4Fun (level 3), the network was clustered into 6 modules (module 1–6, Figure 7D), accounting for 40.21%, 29.18%, 22.06%, 4.63%, 2.14%, and 1.78% of the entire network, respectively. The positive edges accounted for 60.66% of this network. C1, C1%, C2, C2%, and C3% belonged to module 3, whereas C3 belonged to module 5. Furthermore, C1 was significantly related to phenylalanine metabolism (*R* = −0.63, *p* < 0.001), *N*-glycan biosynthesis (*R* = 0.63, *p* < 0.001), pyruvate metabolism (*R* = −0.66, *p* < 0.001), glyoxylate and dicarboxylate metabolism (*R* = −0.63, *p* < 0.001), base excision repair (*R* = 0.60, *p* < 0.01), VEGF signalling pathway (*R* = −0.66, *p* < 0.001), renin angiotensin system (*R* = 0.67, *p* < 0.001), and hematopoietic cell lineage (*R* = 0.67, *p* < 0.001). C1% was related to caffeine metabolism (*R* = −0.61, *p* < 0.01), phenylalanine metabolism (*R* = −0.64, *p* < 0.001), *N*-glycan biosynthesis (*R* = 0.68, *p* < 0.001), glyoxylate and dicarboxylate metabolism (*R* = −0.63, *p* < 0.001), nitrotoluene degradation (*R* = −0.65, *p* < 0.001), base excision repair (*R* = 0.67, *p* < 0.001), renin angiotensin system (*R* = 0.68, *p* < 0.001), and hematopoietic cell lineage (*R* = 0.66, *p* < 0.001). C2 was significantly related to *N*-glycan biosynthesis (*R* = 0.64, *p* < 0.001), pyruvate metabolism (*R* = −0.62, *p* < 0.001), VEGF signalling pathway (*R* = −0.65, *p* < 0.001), renin angiotensin system (*R* = 0.63, *p* < 0.001), and hematopoietic cell lineage (*R* = 0.69, *p* < 0.001). C3 was positively related to cell adhesion molecules (*R* = 0.68, *p* < 0.001), regulation of the actin cytoskeleton (*R* = 0.62, *p* < 0.001), arrhythmogenic right ventricular cardiomyopathy (*R* = 0.64, *p* < 0.001), and dilated cardiomyopathy (*R* = 0.64, *p* < 0.001). C3% was related to phenylalanine metabolism (*R* = 0.61, *p* < 0.01), *N*-glycan biosynthesis (*R* = −0.65, *p* < 0.001), nitrotoluene degradation (*R* = 0.64, *p* < 0.001), base excision repair (*R* = −0.66, *p* < 0.001), lysosome (*R* = −0.62, *p* < 0.01), VEGF signalling pathway (*R* = 0.64, *p* < 0.001), renin angiotensin system (*R* = −0.72, *p* < 0.001), and hematopoietic cell lineage (*R* = −0.70, *p* < 0.001).

For functional composition based on FARPROTAX, the network was clustered into 9 modules (module 1–9, Figure 7E), accounting for 24%, 10%, 10%, 10%, 10%, 10%, 8%, 6%, and 12% of the entire network, respectively. The positive edges accounted for 89.89% in this network. C1, C1%, C2, C2%, and C3% belonged to module 6. Furthermore, C2% was significantly related to aerobic chemoheterotrophy (*R* = 0.61, *p* < 0.01), anoxygenic photoautotrophy *S*-oxidizing (*R* = −0.61, *p* < 0.01), and anoxygenic photoautotrophy (*R* = −0.61, *p* < 0.01).

### 3.8. Potential Drivers of the Water Bacterial Community and Its Functions

**Figure 8:**
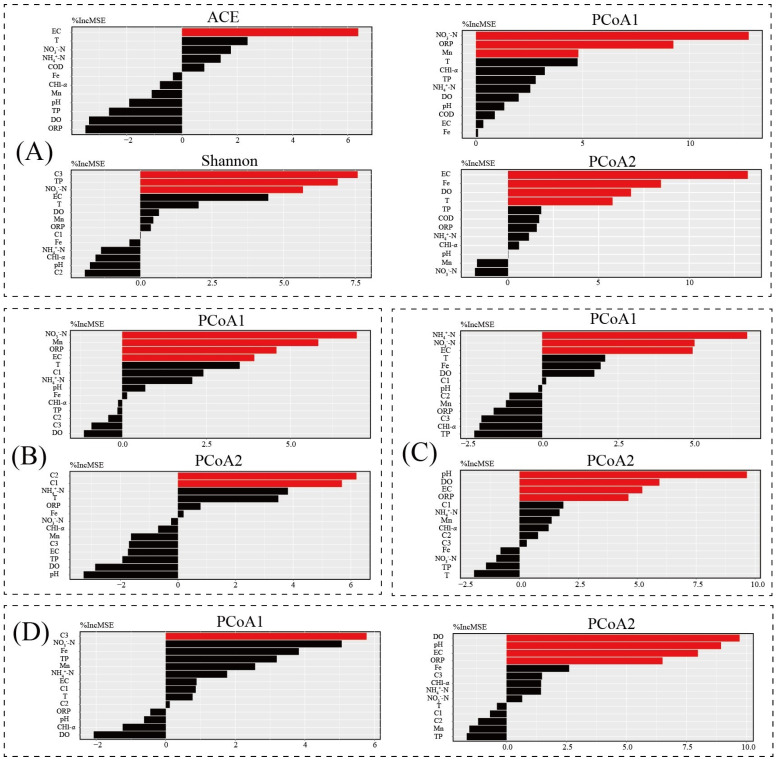
Random Forest analysis is an effective predictor of environment factors as drivers of αdiversity and β-diversity microbial community in Baiyangdian Lake. (**A**) for microbial community; (**B**) for functional community based on PICRUSt2; (**C**) for functional community based on Tax4Fun; (**D**) for functional community based on FARPROTAX.

The main microbial predictors of the water bacterial community and its functional community were determined by RF analysis (*p* < 0.05; Figure 8). For the bacterial community, EC, C3, TP, and NO_3_^−^-N were the most important variables explaining the *α*-diversity of the bacterial community (*p* < 0.05; Figure 8A). NO_3_^−^-N, ORP, and Mn were the most important variables explaining PCoA1 of the bacterial community (*p* < 0.05; Figure 8A). EC, Fe, DO, and T were the most important variables predicting PCoA2 (*p* < 0.05). For the functional composition based on PICRUSt2, NO_3_^−^-N, Mn, ORP, and EC were important variables predicting PCoA1 of the functional community (*p* < 0.05; Figure 8B). C1 and C2 were important variables predicting PCoA2 in the functional community (*p* < 0.05; Figure 8B). For functional composition based on Tax4Fun, NH_4_^+^-N, NO_3_^−^-N, and EC were the most important variables explaining PCoA1 of the bacterial community (*p* < 0.05; Figure 8C). pH, DO, EC, and ORP were the important variables predicting PCoA2 (*p* < 0.05). For functional composition based on FARPROTAX, C3 was an important variable predicting PCoA1 of the functional community (*p* < 0.05; Figure 8D). DO, pH, EC, and ORP were important variables predicting PCoA2 in the functional community (*p* < 0.05; Figure 8D). C1, C2, and C3 were clearly important predictors of *α*- and *β*-diversity of the water bacterial community and its functional composition in ice-covered Baiyangdian Lake. Therefore, it is necessary for managers to understand the interaction between DOM and the microbial community.

## 4. Conclusions

The results showed that the environmental parameters, microbial community, and functional community significantly differed among the typical zones in Baiyangdian Lake, whereas *a*-diversity did not (*p* > 0.05). Moreover, T, pH, ORP, DO, NO_3_^−^-N, NH_4_^+^-N, and Mn were the main environmental factors involved in the shifts of microbial and functional communities based on the RDA and Mantel test. Based on Lefse and STAMP analysis, biomarkers of the microbial community and functional community were enriched in typical zones in Baiyangdian Lake, and most of the top functions were related to carbohydrate metabolism. The results of EEM-PARAFAC revealed two protein-like components (C1 and C2) and one humic-like component (C3); moreover, C1 was significantly correlated with C2 (*R* = 0.99, *p* < 0.001), indicating that they had the same source. The fluorescence intensities and relative abundance significantly differed among the typical zones in Baiyangdian Lake. The high BIX, FI, and β:α and low HIX indicated a strong autochthonous component and aquatic bacterial origin, which was consistent with the results of UV-vis absorption spectroscopy. Correlation network analysis revealed non-random co-occurrence patterns. The bacterial and functional communities interacted closely with CDOM. Thirty-two OTUs were related to C1, 31 OTUs were related to C1%, 33 OTUs were related to C2, 5 OTUs were related to C2%, 25 OTUs were related to C3, and 26 OTUs were related to C3%. The dominant genera were *CL500-29_marine_group*, *Flavobacterium*, *Limnohabitans*, and *Candidatus_Aquirestis*, which were closely related to carbon metabolism. Furthermore, random forest analysis showed that C3 was an important variable explaining the *α*-diversity of the bacterial community; C1 and C2 were the important variables predicting PCoA2 of the functional community based on PICRUSt2; and C3 was an important variable predicting the PCoA1 of functional communities based on FARPROTAX, in ice-covered Baiyangdian Lake. This study provides a method for investigating the interaction between bacterial communities and DOM in ice-covered Baiyangdian Lake.

## Figures and Tables

**Table 1:** Community variances explained by environmental factors of microbial community and function community, respectively, in Baiyangdian Lake.

		VIF	RDA1	RDA2	R^2^	*p*
Taxonomic composition (F = 2.156, *p* <0.001)	T	9.66	0.98	0.21	0.49	0.003
	pH	1.92	−0.78	−0.63	0.26	0.039
	ORP	2.09	−0.97	0.23	0.12	0.227
	EC	4.2	0.29	0.96	0.65	0.001
	DO	6.61	−0.48	−0.88	0.41	0.004
	CHl-α	3.3	−0.59	−0.81	0.02	0.785
	NO_3_^−^-N	6.48	−0.95	0.3	0.87	0.001
	NH_4_^+^-N	4.39	−0.97	0.24	0.28	0.036
	TP	2.01	−0.80	0.61	0.29	0.026
	Fe	1.68	−0.57	−0.82	0.31	0.019
	Mn	1.98	−0.99	0.13	0.38	0.013
Functional composition based on PICRUSt2 (F = 3.798, *p* < 0.001)	T	8.88	0.89	0.46	0.48	0.003
	ORP	2.15	−0.82	0.57	0.2	0.123
	EC	3.9	0.81	0.58	0.13	0.206
	DO	5.47	−0.78	−0.63	0.1	0.325
	NO_3_^−^-N	4.88	−1.00	−0.05	0.56	0.001
	NH_4_^+^-N	6.33	−0.97	−0.24	0.33	0.02
	TP	2.02	−0.96	0.29	0.22	0.078
	COD_Mn_	2.09	0.99	−0.16	0.07	0.488
	Fe	1.68	−0.36	−0.93	0.39	0.008
	Mn	1.86	−0.99	−0.11	0.47	0.001
Functional composition based on Tax4Fun (F = 1.922, *p* < 0.05)	pH	1.81	−0.17	−0.99	0.3	0.022
	DO	1.76	−0.58	−0.81	0.37	0.007
	CHl-α	2.37	−0.55	−0.83	0.13	0.236
	NO_3_^−^-N	3.02	−0.99	−0.16	0.14	0.194
	NH_4_^+^-N	2.89	−0.59	0.81	0.23	0.069
	TP	1.66	−0.90	0.43	0.12	0.241
	COD_Mn_	1.68	0.2	0.98	0.2	0.087
	Fe	1.35	−0.98	−0.19	0.11	0.298
	Mn	1.87	−0.17	0.99	0.09	0.365
Functional composition based on FARPROTAX (F = 1.533, *p* < 0.05)	T	10.94	−0.31	0.95	0.22	0.068
	pH	1.99	0.71	−0.71	0.51	0.001
	EC	3.86	−0.81	0.59	0.41	0.006
	DO	6.8	0.77	−0.64	0.43	0.003
	CHl-α	3.73	0.51	−0.86	0.1	0.314
	NO_3_^−^-N	4.69	−0.28	−0.96	0.48	0.003
	NH_4_^+^-N	5.12	−0.88	−0.48	0.15	0.159
	TP	1.95	−0.84	−0.54	0.25	0.043
	COD_Mn_	2.37	−0.19	0.98	0.15	0.146
	Fe	1.47	0.88	−0.48	0.13	0.214
	Mn	1.9	−0.38	−0.93	0.34	0.018

**Table 2:** Spectral characteristics of the three components identified by PARAFAC analysis in this study and their comparison with previously identified components.

Components	Ex/Em	Description and Source Assignment	Reference
C1	275/325	Protein-like substance	275/330 (Li et al., 2020); 275/340 (Ziegmann et al., 2010)
C2	225/345	Tryptophan-like DOM	230/355 (Li et al., 2020); 230/330 (Stedmon et al., 2003)
C3	250/410	Humic-like Substance (UVC)	240/415 (Cory and Mcknight, 2005; Stedmon et al., 2003); 260(355)/434 (Murphy et al., 2008)

**Table 3:** Topological properties of the co-occurrence networks of microbial communities in Bauyangdian Lake.

	Empirical Network				Random Network			
	Type1	Type2	Type3	Type4	Type1	Type2	Type3	Type4
Nodes	471	373	281	50	471	373	281	50
Edges	3962	12470	7659	89	3962	12470	7659	89
Modularity	0.43	0.234	0.363	0.68	0.205 ± 0.004	0.205 ± 0.005	0.205 ± 0.005	0.205 ± 0.009
Clustering coefficient	0.38	0.69	0.64	0.61	0.036 ± 0.001	0.036 ± 0.005	0.036 ± 0.005	0.036 ± 0.001
Network diameter	5.72	5.63	3.91	6.01	4.000 ± 0.032	4.000 ± 0.063	4.000 ± 0.032	4.000 ± 0.063
Average path length	3.14	2.33	2.14	3.30	2.493 ± 0.001	2.493 ± 0.021	2.493 ± 0.021	2.494 ± 0.024
Closeness centrality	0.17	0.005	0.18	0.023	0.090 ± 0.011	0.090 ± 0.010	0.090 ± 0.010	0.090 ± 0.011
Network density	0.04	0.180	0.195	0.073	0.036 ± 0.000	0.036 ± 0.005	0.036 ± 0.005	0.036 ± 0.000
Betweenness centrality	0.03	0.04	0.019	0.16	0.007 ± 0.001	0.007 ± 0.001	0.007 ± 0.001	0.007 ± 0.005
Degree centralization	0.09	0.27	0.17	0.13	0.028 ± 0.004	0.029 ± 0.004	0.029 ± 0.004	0.028 ± 0.004

Type1, network based on OTU-DOM; Type2, network based on Function composition (PICRUSt2)-DOM; Type3, network based on Function composition (Tax4Fun)-DOM; Type4, network based on Function composition (FARPROTAX)-DOM.

## Supplementary Materials

Supplementary materials can be found at https://www.mdpi.com/2076-2607/8/6/883/s1.
