# Supplementary material for: Linking Shifts in Bacterial Community Composition and Function with Changes in the Dissolved Organic Matter Pool in Ice-Covered Baiyangdian Lake, Northern China
Source: Microorganisms. 2020 Jun 11;8(6):883. doi: 10.3390/microorganisms8060883 (PMC7357102; doi:10.3390/microorganisms8060883)
Supplement: Supplementary file 1 [file microorganisms-08-00883-s001.pdf]

2.3.1. Fluorescence analyses

Fluorescence excitation–emission matrix (EEM) spectroscopy was performed using a F-7000 fluorescence spectrophotometer (Japan) in a clear quartz curette. Excitation and emission were simultaneously scanned at wavelengths ranging from 200 to 450 nm at 5-nm intervals and from 250 to 600 nm, at 1-nm intervals, respectively. The slit width was 10 nm for both excitation and emission monochromators and the scan speed was set to 2400 nm min^{-1}. The corrected and standardized EEMs were modeled using PARAFAC analysis. The PARAFAC analysis in this work was performed in MATLAB using the DOMFluor toolbox (http://www.models.kvl.dk/), according to the procedure recommended in Stedmon and Bro (2008) (Stedmon and Bro, 2008). In this study, in order to minimize the differences in scores, the fluorescence measurements were performed at the same temperature (25 ± 1 °C). Meanwhile, EEMs are presented in Raman Units (R.U.) (Lawaetz and Stedmon, 2009).

2.3.2 Fluorescence spectroscopy analyses

According to Lavonen et al., (Lavonen et al., 2015), the fluorescence index (FI) is a proxy of sources of fulvic acids, which represents the ratio of the emission intensity at a wavelength of 470 to that at 520 nm, with a calculated excitation wavelength of 370 nm; and the biological index (BIX) (Huguet et al., 2009), an index of recent autochthonous and biological contribution, is a ratio of the emission wavelength at 380 and 430 nm when the excitation wavelength is 310 nm; and the humification index (HIX) (Ohno, 2002), is a ratio of the areas under the emission spectra over 435–480 nm to that by the sum of the area at emission of 300 to 345 nm at an Ex of 254 nm; the freshness index (β:α) was calculated as the ratio of emission at 380 nm divided by the emission maximum between 420 and 435, at an excitation of 310 nm (Zhou et al., 2019). *F*n280 and *F*n355 represented for the relative abundance of protein-like substance and humic-like substance (Zhang et al., 2009).

2.3.3 UV–Visible absorption spectroscopy analyses

The UV–Visible absorbance spectra on each extracted sample were performed by using a UV–Vis spectrometer (DR6000) from 200 to 800 nm and using Millipore water as blank with 1 cm quartz cuvette (Shafiquzzaman et al., 2014). The specific UV absorbance at 254 nm (α_{254}) and at 355 nm (α_{355}) were acted as an indicator of DOM concentration (Zhou et al., 2019). The ratio of absorption at 250 nm to 365 nm (E2/E3) was calculated as a proxy of the aromaticity and the average molecular weight of DOM (Li and Hur, 2017). The E3/E4 ratio, an indicator of aromaticity of aquatic humic substances of DOM, was calculated by dividing the absorbance at 300 nm by the absorbance at 400 nm (Li and Hur, 2017). Absorption spectral slope ratio (S_R, the ratio of log transformed absorbance spectra slope at 275–295 nm to that of 350–400 nm) is inversely correlated to the Molecular weight (MW) of DOM (Helms et al., 2008). The E4/E6 was related to the degree of humic (Chen et al., 1977). More detailed information of UV–Visible absorption spectroscopy analysis has been described elsewhere (Li and Hur, 2017).

Figure lengend

Figure S1. Relative abundance of bacterial communities in Baiyangdian Lake. (A, at the phylum level; B, at the class level; C, at the genus level)

Figure S2. PCoA of microbial structure in Baiyangdian Lake.

Figure S3. The relative abundance of various predicted functions of microbial communities based on PICRUSt2, Tax4Fun, and FARPROTAX in Baiyangdian Lake.

Figure S4. Ultraviolet-visible spectroscopy, fluorescence intensity and relative abundance, and correlations of spectral characteristic and environment factors based on UV-vis and EEMs in Baiyangdian Lake. (A, Ultraviolet-visible spectroscopy; B, fluorescence intensity and relative abundance; C, correlations of spectral characteristic and environment factors based on UV-vis; D, correlations of spectral characteristic and environment factors based on EEMs)

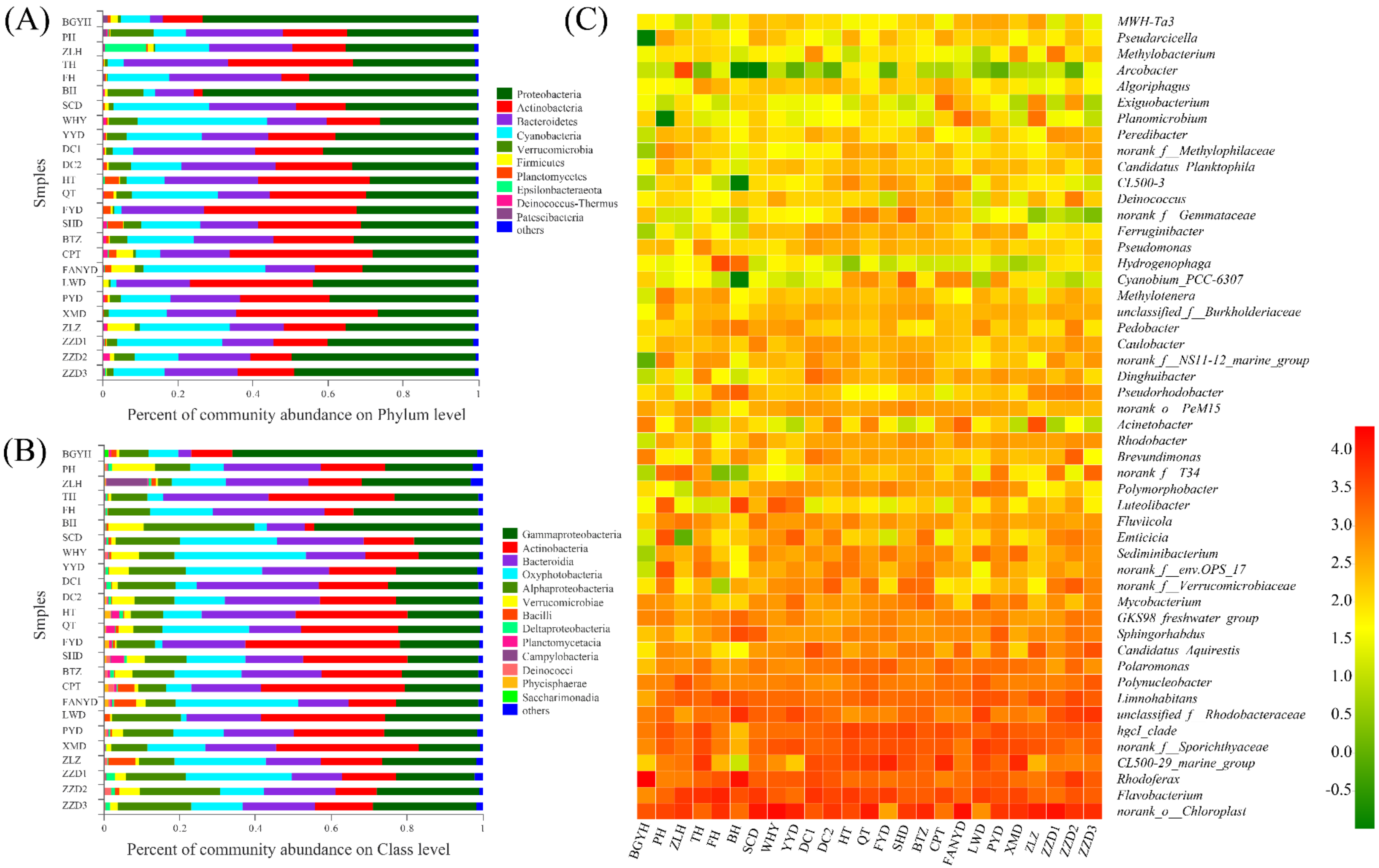
(A)
BGYH
PH
ZLH
TH
FH
BH
SCD
WHY
YYD
DC1
DC2
HT
QT
FYD
SHD
BTZ
CPT
FANYD
LWD
PYD
XMD
ZLZ
ZZD1
ZZD2
ZZD3
Smples
0
0.2
0.4
0.6
0.8
1
Percent of community abundance on Phylum level
Proteobacteria
Actinobacteria
Bacteroidetes
Cyanobacteria
Verrucomicrobia
Firmicutes
Planctomycetes
Epsilonbacteraeota
Deinococcus-Thermus
Patescibacteria
others
(B)
BGYH
PH
ZLH
TH
FH
BH
SCD
WHY
YYD
DC1
DC2
HT
QT
FYD
SHD
BTZ
CPT
FANYD
LWD
PYD
XMD
ZLZ
ZZD1
ZZD2
ZZD3
Smples
0
0.2
0.4
0.6
0.8
1
Percent of community abundance on Class level
Gammaproteobacteria
Actinobacteria
Bacteroidia
Oxyphotobacteria
Alphaproteobacteria
Verrucomicrobiae
Bacilli
Deltaproteobacteria
Planctomycetacia
Campylobacteria
Deinococci
Phycisphaerae
Saccharimonadia
others
(C)
MWH-Ta3
Pseudarcicella
Methylobacterium
Arcobacter
Algoriphagus
Exiguobacterium
Planomicrobium
Peredibacter
norank_f__Methylophilaceae
Candidatus_Planktophila
CL500-3
Deinococcus
norank_f__Gemmataceae
Ferruginibacter
Pseudomonas
Hydrogenophaga
Cyanobium_PCC-6307
Methylotenera
unclassified_f__Burkholderiaceae
Pedobacter
Caulobacter
norank_f__NS11-12_marine_group
Dinghuibacter
Pseudorhodobacter
norank_o__PeM15
Acinetobacter
Rhodobacter
Brevundimonas
norank_f__T34
Polymorphobacter
Luteolibacter
Fluviicola
Emticicia
Sediminibacterium
norank_f__env.OPS_17
norank_f__Verrucomicrobiaceae
Mycobacterium
GKS98_freshwater_group
Sphingorhabdus
Candidatus_Aquirestis
Polaromonas
Polynucleobacter
Limnohabitans
unclassified_f__Rhodobacteraceae
hgcI_clade
norank_f__Sporichthyaceae
CL500-29_marine_group
Rhodoferax
Flavobacterium
norank_o__Chloroplast
BGYH
PH
ZLH
TH
FH
BH
SCD
WHY
YYD
DC1
DC2
HT
QT
FYD
SHD
BTZ
CPT
FANYD
LWD
PYD
XMD
ZLZ
ZZD1
ZZD2
ZZD3
4.0
3.5
3.0
2.5
2.0
1.5
1.0
0.5
0.0
-0.5

Figure S1

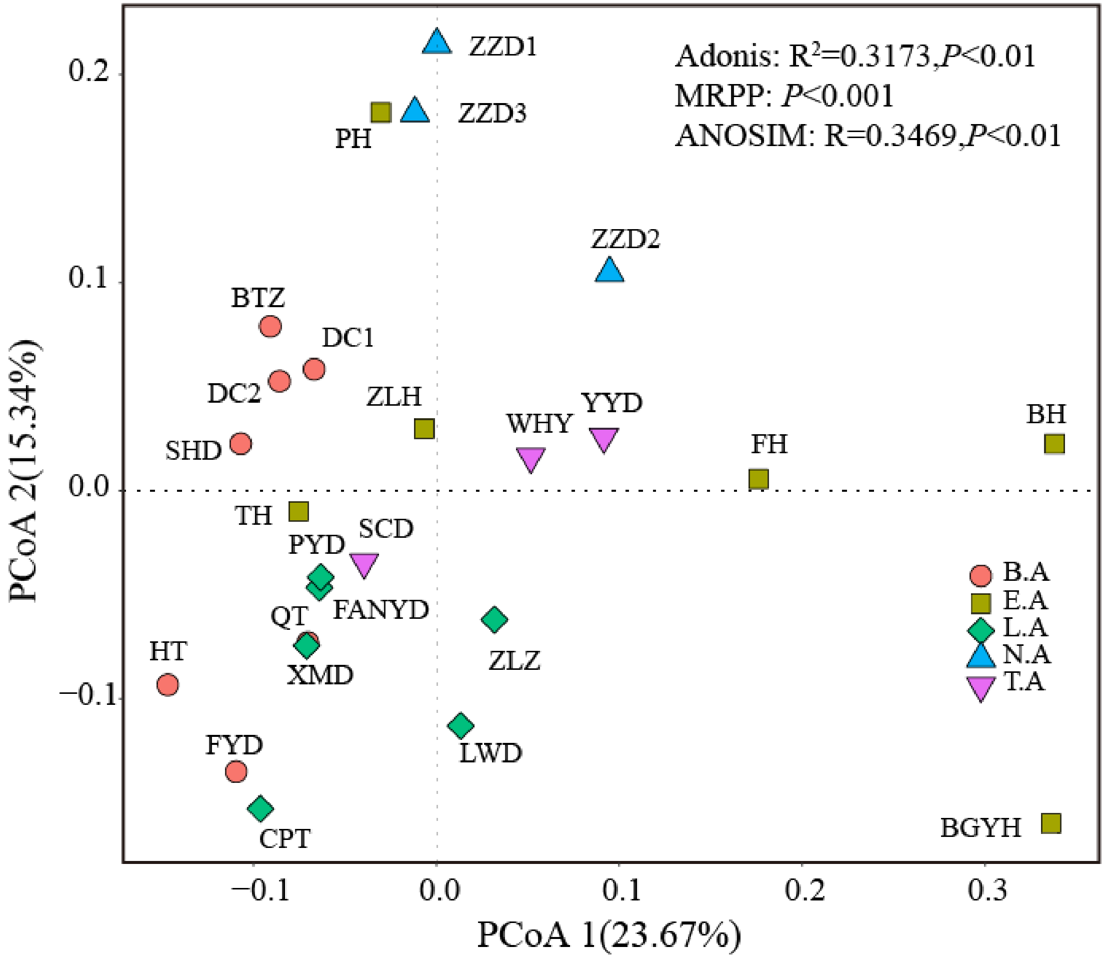

Adonis: R²=0.3173,P<0.01
MRPP: P<0.001
ANOSIM: R=0.3469,P<0.01
ZZD1
ZZD3
PH
ZZD2
BTZ
DC1
DC2
ZLH
WHY
YYD
FH
BH
SHD
TH
PYD
SCD
QT
FANYD
ZLZ
HT
XMD
FYD
LWD
CPT
BGYH
B.A
E.A
L.A
N.A
T.A
0.2
0.1
0.0
−0.1
−0.1
0.0
0.1
0.2
0.3
PCoA 1(23.67%)
PCoA 2(15.34%)

Figure S2

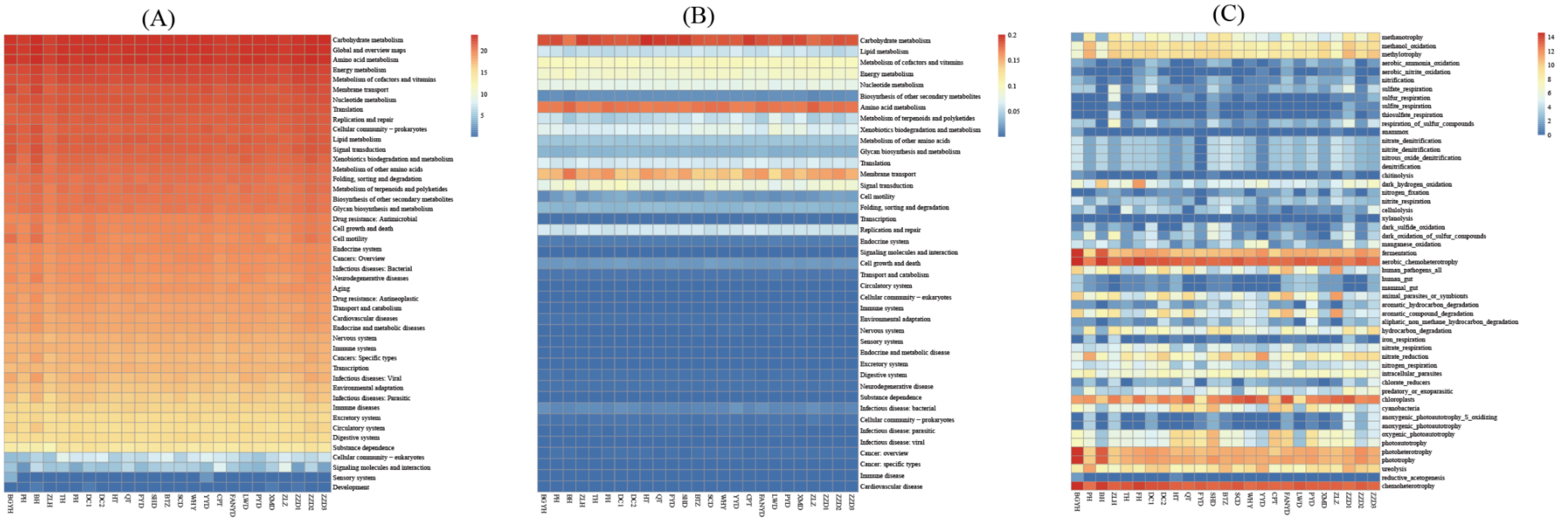

(A)
Carbohydrate metabolism
Global and overview maps
Amino acid metabolism
Energy metabolism
Metabolism of cofactors and vitamins
Membrane transport
Nucleotide metabolism
Translation
Replication and repair
Cellular community − prokaryotes
Lipid metabolism
Signal transduction
Xenobiotics biodegradation and metabolism
Metabolism of other amino acids
Folding, sorting and degradation
Metabolism of terpenoids and polyketides
Biosynthesis of other secondary metabolites
Glycan biosynthesis and metabolism
Drug resistance: Antimicrobial
Cell growth and death
Cell motility
Endocrine system
Cancers: Overview
Infectious diseases: Bacterial
Neurodegenerative diseases
Aging
Drug resistance: Antineoplastic
Transport and catabolism
Cardiovascular diseases
Endocrine and metabolic diseases
Nervous system
Immune system
Cancers: Specific types
Transcription
Infectious diseases: Viral
Environmental adaptation
Infectious diseases: Parasitic
Immune diseases
Excretory system
Circulatory system
Digestive system
Substance dependence
Cellular community − eukaryotes
Signaling molecules and interaction
Sensory system
Development
20
15
10
5
BGYH
PH
BH
ZLH
TH
FH
DC1
DC2
HT
QT
PYD
SHD
BTZ
SCD
WHY
YYD
CPT
FANYD
LWD
PYD
XMD
ZLZ
ZZD1
ZZD2
ZZD3
(B)
Carbohydrate metabolism
Lipid metabolism
Metabolism of cofactors and vitamins
Energy metabolism
Nucleotide metabolism
Biosynthesis of other secondary metabolites
Amino acid metabolism
Metabolism of terpenoids and polyketides
Xenobiotics biodegradation and metabolism
Metabolism of other amino acids
Glycan biosynthesis and metabolism
Translation
Membrane transport
Signal transduction
Cell motility
Folding, sorting and degradation
Transcription
Replication and repair
Endocrine system
Signaling molecules and interaction
Cell growth and death
Transport and catabolism
Circulatory system
Cellular community − eukaryotes
Immune system
Environmental adaptation
Nervous system
Sensory system
Endocrine and metabolic disease
Excretory system
Digestive system
Neurodegenerative disease
Substance dependence
Infectious disease: bacterial
Cellular community − prokaryotes
Infectious disease: parasitic
Infectious disease: viral
Cancer: overview
Cancer: specific types
Immune disease
Cardiovascular disease
0.2
0.15
0.1
0.05
BGYH
PH
BH
ZLH
TH
FH
DC1
DC2
HT
QT
PYD
SHD
BTZ
SCD
WHY
YYD
CPT
FANYD
LWD
PYD
XMD
ZLZ
ZZD1
ZZD2
ZZD3
(C)
methanotrophy
methanol_oxidation
methylotrophy
aerobic_ammonia_oxidation
aerobic_nitrite_oxidation
nitrification
sulfate_respiration
sulfur_respiration
sulfite_respiration
thiosulfate_respiration
respiration_of_sulfur_compounds
anammox
nitrate_denitrification
nitrite_denitrification
nitrous_oxide_denitrification
denitrification
chitinolysis
dark_hydrogen_oxidation
nitrogen_fixation
nitrite_respiration
cellulolysis
xylanolysis
dark_sulfide_oxidation
dark_oxidation_of_sulfur_compounds
manganese_oxidation
fermentation
aerobic_chemoheterotrophy
human_pathogens_all
human_gut
mammal_gut
animal_parasites_or_symbionts
aromatic_hydrocarbon_degradation
aromatic_compound_degradation
aliphatic_non_methane_hydrocarbon_degradation
hydrocarbon_degradation
iron_respiration
nitrate_respiration
nitrate_reduction
nitrogen_respiration
intracellular_parasites
chlorate_reducers
predatory_or_exoparasitic
chloroplasts
cyanobacteria
anoxygenic_photoautotrophy_S_oxidizing
anoxygenic_photoautotrophy
oxygenic_photoautotrophy
photoautotrophy
photoheterotrophy
phototrophy
ureolysis
reductive_acetogenesis
chemoheterotrophy
14
12
10
8
6
4
2
0
BGYH
PH
BH
ZLH
TH
FH
DC1
DC2
HT
QT
PYD
SHD
BTZ
SCD
WHY
YYD
CPT
FANYD
LWD
PYD
XMD
ZLZ
ZZD1
ZZD2
ZZD3

Figure S3

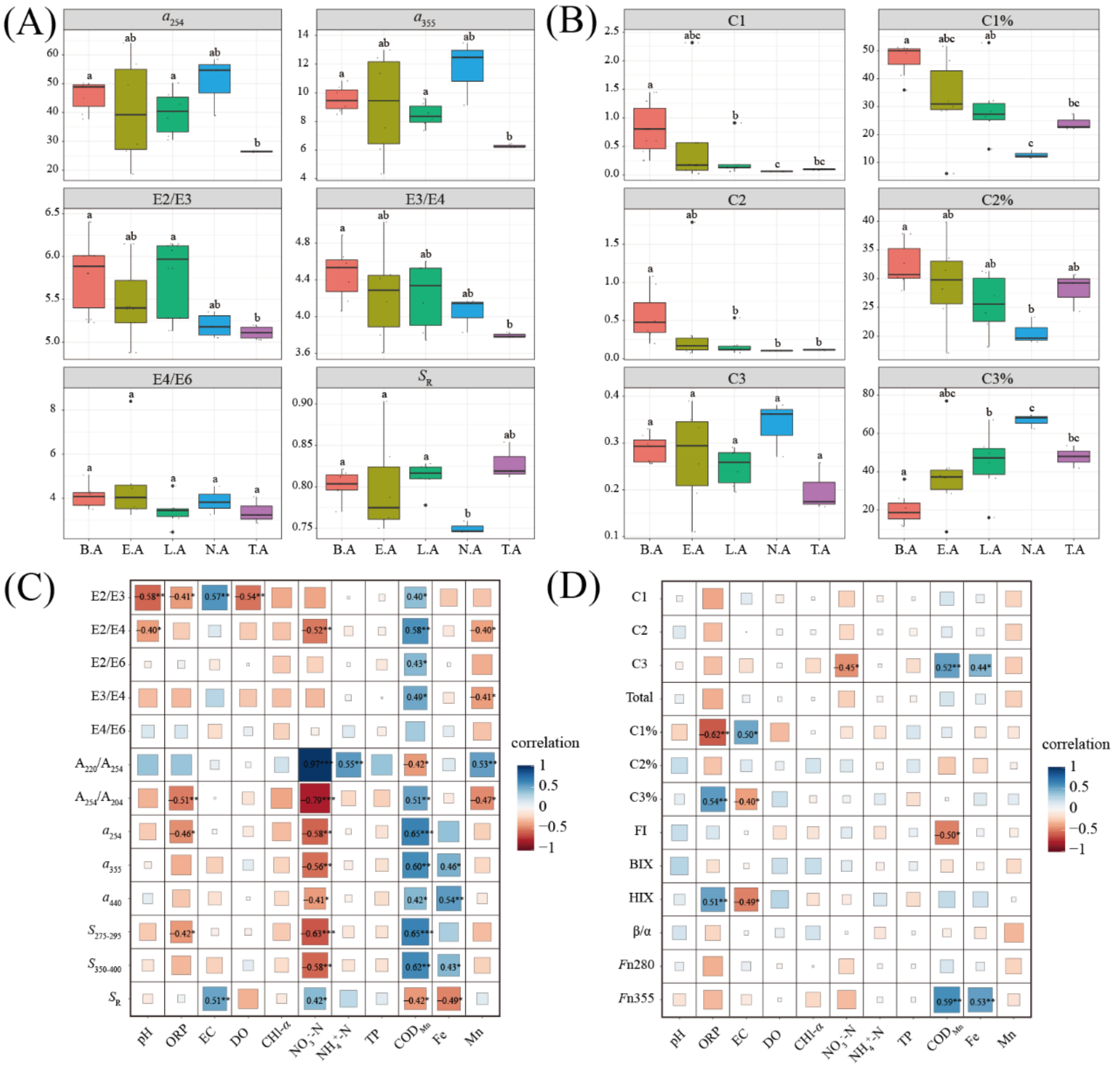
(A)
(B)
(C)
(D)
E2/E3
E3/E4
E4/E6
C1
C2
C3
C1%
C2%
C3%
B.A
E.A
L.A
N.A
T.A
E2/E4
E2/E6
Total
FI
BIX
HIX
β/α
Fn280
Fn355
correlation
pH
ORP
EC
DO
TP
Fe
Mn

Figure S4

Table S1 Topological properties of microbial community related to C1 in Bauyangdian Lake.

Nodes	Phylum	Genus	Modularity	Degree	Closness centrality	Betweenness centrality	Clustering
OTU5141	Proteobacteria	*unclassified Sphingomonadaceae*	2	8	0.30	40.23	0.46
OTU3359	Actinobacteria	*Unclassified Actinobacteria*	2	44	0.38	799.07	0.39
OTU5117	Proteobacteria	*Roseomonas*	2	12	0.33	251.78	0.36
OTU1301	Cyanobacteria	*Planktothrix_NIVA-CYA_15*	2	49	0.39	1077.52	0.37
OTU9975	Patescibacteria	*norank Saccharimonadales*	2	15	0.34	212.41	0.42
OTU4613	Actinobacteria	*Norank PeM15*	2	21	0.34	492.61	0.49
OTU1988	Actinobacteria	*Norank Gaiellales*	2	48	0.38	1053.17	0.40
OTU8195	Cyanobacteria	*Norank Chloroplast*	2	21	0.34	139.84	0.60
OTU4334	Cyanobacteria	*Norank Chloroplast*	2	38	0.38	718.37	0.43
OTU1619	Cyanobacteria	*Norank Chloroplast*	2	42	0.37	640.22	0.45
OTU10572	Cyanobacteria	*Norank Chloroplast*	2	21	0.35	131.76	0.54
OTU1622	Actinobacteria	*Norank Sporichthyaceae*	2	20	0.35	142.49	0.56
OTU8159	Proteobacteria	*Norank Mitochondria*	4	31	0.36	968.69	0.33
OTU1043	Planctomycetes	*Norank Gemmataceae*	2	35	0.35	683.95	0.47
OTU6234	Bacteroidetes	*Norank Chitinophagaceae*	2	27	0.36	377.05	0.46
OTU3340	Proteobacteria	*MWH-UniP1_aquatic_group*	4	47	0.37	701.58	0.40
OTU9287	Verrucomicrobia	*Luteolibacter*	2	12	0.32	332.18	0.45
OTU8189	Verrucomicrobia	*Luteolibacter*	2	38	0.39	1177.65	0.37
OTU7082	Actinobacteria	*hgcI_clade*	2	49	0.37	763.34	0.41
OTU2316	Bacteroidetes	*Fluviicola*	2	26	0.36	732.15	0.43
OTU7100	Bacteroidetes	*Flavobacterium*	2	39	0.38	1053.93	0.43
OTU6236	Bacteroidetes	*Flavobacterium*	2	33	0.37	533.55	0.45
OTU2247	Bacteroidetes	*Flavobacterium*	2	10	0.31	46.96	0.78
OTU2246	Bacteroidetes	*Flavobacterium*	2	30	0.38	876.93	0.48
OTU3358	Bacteroidetes	*Dinghuibacter*	2	41	0.37	663.75	0.44
OTU3640	Cyanobacteria	*Cyanobium_PCC-6307*	2	42	0.38	639.69	0.45
OTU4279	Planctomycetes	*CL500-3*	2	32	0.37	286.80	0.48
OTU2721	Actinobacteria	*CL500-29_marine_group*	2	59	0.40	1643.05	0.35
OTU1748	Actinobacteria	*CL500-29_marine_group*	2	54	0.38	781.67	0.42
OTU1630	Actinobacteria	*CL500-29_marine_group*	2	40	0.37	456.93	0.46
OTU5491	Actinobacteria	*Candidatus Limnoluna*	2	38	0.37	471.64	0.43
OTU3354	Actinobacteria	*Aurantimicrobium*	2	36	0.36	689.99	0.40

Table S2 Topological properties of microbial community related to C1% in Bauyangdian Lake.

Nodes	Phylum	Genus	Modularity	Degree	Closness centrality	Betweenness centrality	Clustering
OTU3359	Actinobacteria	*unclassified_c__Actinobacteria*	2	44	0.38	799.07	0.39
OTU5117	Proteobacteria	*Roseomonas*	2	12	0.33	251.78	0.36
OTU4318	Actinobacteria	*norank_o__PeM15*	3	17	0.33	660.73	0.35
OTU4613	Actinobacteria	*norank_o__PeM15*	2	21	0.34	492.61	0.49
OTU1988	Actinobacteria	*norank_o__Gaiellales*	2	48	0.38	1053.17	0.40
OTU5112	Cyanobacteria	*norank_o__Chloroplast*	3	20	0.34	486.45	0.51
OTU5759	Cyanobacteria	*norank_o__Chloroplast*	3	17	0.34	319.60	0.56
OTU10572	Cyanobacteria	*norank_o__Chloroplast*	2	21	0.35	131.76	0.54
OTU1619	Cyanobacteria	*norank_o__Chloroplast*	2	42	0.37	640.22	0.45
OTU4334	Cyanobacteria	*norank_o__Chloroplast*	2	38	0.38	718.37	0.43
OTU8195	Cyanobacteria	*norank_o__Chloroplast*	2	21	0.34	139.84	0.60
OTU1622	Actinobacteria	*norank_f__Sporichthyaceae*	2	20	0.35	142.49	0.56
OTU8159	Proteobacteria	*norank_f__Mitochondria*	4	31	0.36	968.69	0.33
OTU1043	Planctomycetes	*norank_f__Gemmataceae*	2	35	0.35	683.95	0.47
OTU9287	Verrucomicrobia	*Luteolibacter*	2	12	0.32	332.18	0.45
OTU7082	Actinobacteria	*hgcI_clade*	2	49	0.37	763.34	0.41
OTU2316	Bacteroidetes	*Fluviicola*	2	26	0.36	732.15	0.43
OTU10548	Bacteroidetes	*Flavobacterium*	2	24	0.36	635.82	0.51
OTU2246	Bacteroidetes	*Flavobacterium*	2	30	0.38	876.93	0.48
OTU2247	Bacteroidetes	*Flavobacterium*	2	10	0.31	46.96	0.78
OTU6236	Bacteroidetes	*Flavobacterium*	2	33	0.37	533.55	0.45
OTU7100	Bacteroidetes	*Flavobacterium*	2	39	0.38	1053.93	0.43
OTU9951	Bacteroidetes	*Emticicia*	3	10	0.34	529.29	0.09
OTU3358	Bacteroidetes	*Dinghuibacter*	2	41	0.37	663.75	0.44
OTU3640	Cyanobacteria	*Cyanobium_PCC-6307*	2	42	0.38	639.69	0.45
OTU4279	Planctomycetes	*CL500-3*	2	32	0.37	286.80	0.48
OTU1630	Actinobacteria	*CL500-29_marine_group*	2	40	0.37	456.93	0.46
OTU1748	Actinobacteria	*CL500-29_marine_group*	2	54	0.38	781.67	0.42
OTU2721	Actinobacteria	*CL500-29_marine_group*	2	59	0.40	1643.05	0.35
OTU5491	Actinobacteria	*Candidatus_Limnoluna*	2	38	0.37	471.64	0.43
OTU3354	Actinobacteria	*Aurantimicrobium*	2	36	0.36	689.99	0.40

Table S3 Topological properties of microbial community related to C2 in Bauyangdian Lake.

Nodes	Phylum	Genus	Modularity	Degree	Closness centrality	Betweenness centrality	Clustering
OTU1011	Bacteroidetes	*Ferruginibacter*	2	47	0.39	2050.98	0.26
OTU1043	Planctomycetes	*norank_f__Gemmataceae*	2	35	0.35	683.95	0.47
OTU10548	Bacteroidetes	*Flavobacterium*	2	24	0.36	635.82	0.51
OTU10572	Cyanobacteria	*norank_o__Chloroplast*	2	21	0.35	131.76	0.54
OTU1619	Cyanobacteria	*norank_o__Chloroplast*	2	42	0.37	640.22	0.45
OTU1622	Actinobacteria	*norank_f__Sporichthyaceae*	2	20	0.35	142.49	0.56
OTU1630	Actinobacteria	*CL500-29_marine_group*	2	40	0.37	456.93	0.46
OTU1748	Actinobacteria	*CL500-29_marine_group*	2	54	0.38	781.67	0.42
OTU1988	Actinobacteria	*norank_o__Gaiellales*	2	48	0.38	1053.17	0.40
OTU2246	Bacteroidetes	*Flavobacterium*	2	30	0.38	876.93	0.48
OTU2247	Bacteroidetes	*Flavobacterium*	2	10	0.31	46.96	0.78
OTU2316	Bacteroidetes	*Fluviicola*	2	26	0.36	732.15	0.43
OTU2721	Actinobacteria	*CL500-29_marine_group*	2	59	0.40	1643.05	0.35
OTU3354	Actinobacteria	*Aurantimicrobium*	2	36	0.36	689.99	0.40
OTU3358	Bacteroidetes	*Dinghuibacter*	2	41	0.37	663.75	0.44
OTU3359	Actinobacteria	*unclassified_c__Actinobacteria*	2	44	0.38	799.07	0.39
OTU3640	Cyanobacteria	*Cyanobium_PCC-6307*	2	42	0.38	639.69	0.45
OTU3700	Bacteroidetes	*Flavobacterium*	2	17	0.34	862.09	0.38
OTU4279	Planctomycetes	*CL500-3*	2	32	0.37	286.80	0.48
OTU4334	Cyanobacteria	*norank_o__Chloroplast*	2	38	0.38	718.37	0.43
OTU4613	Actinobacteria	*norank_o__PeM15*	2	21	0.34	492.61	0.49
OTU5117	Proteobacteria	*Roseomonas*	2	12	0.33	251.78	0.36
OTU5491	Actinobacteria	*Candidatus_Limnoluna*	2	38	0.37	471.64	0.43
OTU6234	Bacteroidetes	*norank_f__Chitinophagaceae*	2	27	0.36	377.05	0.46
OTU6236	Bacteroidetes	*Flavobacterium*	2	33	0.37	533.55	0.45
OTU7082	Actinobacteria	*hgcI_clade*	2	49	0.37	763.34	0.41
OTU7100	Bacteroidetes	*Flavobacterium*	2	39	0.38	1053.93	0.43
OTU7831	Bacteroidetes	*Dinghuibacter*	2	36	0.39	2052.59	0.24
OTU8189	Verrucomicrobia	*Luteolibacter*	2	38	0.39	1177.65	0.37
OTU8195	Cyanobacteria	*norank_o__Chloroplast*	2	21	0.34	139.84	0.60
OTU9287	Verrucomicrobia	*Luteolibacter*	2	12	0.32	332.18	0.45
OTU9975	Patescibacteria	*norank_o__Saccharimonadales*	2	15	0.34	212.41	0.42

OTU4670	Bacteroidetes	Flavobacterium	4	8	0.31	366.68	0.25

Table S4 Topological properties of microbial community related to C2% in Bauyangdian Lake.

Nodes	Phylum	Genus	Modularity	Degree	Closness centrality	Betweenness centrality	Clustering
OTU1988	Actinobacteria	*norank_o__Gaiellales*	2	48	0.38	1053.17	0.40
OTU5117	Proteobacteria	*Roseomonas*	2	12	0.33	251.78	0.36
OTU9287	Verrucomicrobia	*Luteolibacter*	2	12	0.32	332.18	0.45
OTU5071	Proteobacteria	*Limnohabitans*	3	5	0.30	587.88	0.10
OTU3874	Epsilonbacteraeota	*Sulfuricurvum*	1	6	0.30	141.26	0.20

Table S5 Topological properties of microbial community related to C3 in Bauyangdian Lake.

Nodes	Phylum	Genus	Modularity	Degree	Closness centrality	Betweenness centrality	Clustering
OTU9992	Proteobacteria	*unclassified_p__Proteobacteria*	1	19	0.35	160.79	0.52
OTU7828	Bacteroidetes	*unclassified_c__Bacteroidia*	1	35	0.37	459.00	0.52
OTU9974	Proteobacteria	*Rhodoferax*	1	36	0.37	1155.65	0.35
OTU10024	Proteobacteria	*Rheinheimera*	1	44	0.39	969.85	0.43
OTU2001	Proteobacteria	*Peredibacter*	1	53	0.41	1331.26	0.34
OTU10564	Proteobacteria	*OM27_clade*	1	28	0.37	416.66	0.43
OTU10558	Cyanobacteria	*norank_o__Chloroplast*	1	37	0.40	1104.80	0.38
OTU10578	Cyanobacteria	*norank_o__Chloroplast*	1	18	0.34	124.71	0.44
OTU9943	Cyanobacteria	*norank_o__Chloroplast*	1	41	0.39	1119.33	0.35
OTU1036	Bacteroidetes	*norank_o__Chitinophagales*	1	33	0.37	405.39	0.46
OTU10007	Proteobacteria	*norank_f__T34*	1	16	0.34	221.46	0.40
OTU10023	Proteobacteria	*norank_f__T34*	1	40	0.39	1251.08	0.38
OTU3689	Proteobacteria	*norank_f__T34*	1	46	0.40	1296.81	0.36
OTU8598	Proteobacteria	*norank_f__T34*	1	25	0.38	1769.65	0.28
OTU9891	Proteobacteria	*norank_f__Neisseriaceae*	1	42	0.38	1284.04	0.38
OTU5135	Proteobacteria	*norank_f__Mitochondria*	1	12	0.32	137.95	0.33
OTU8625	Bacteroidetes	*norank_f__Chitinophagaceae*	1	51	0.40	1560.86	0.34
OTU5139	Proteobacteria	*Methylobacter*	1	48	0.39	1603.68	0.34
OTU6011	Bacteroidetes	*Lutibacter*	1	3	0.32	11.09	0.33
OTU3346	Spirochaetes	*Leptospira*	1	27	0.37	728.62	0.41
OTU1777	Bacteroidetes	*Flavobacterium*	1	31	0.37	575.02	0.38
OTU4274	Actinobacteria	*CL500-29_marine_group*	1	18	0.34	1078.32	0.23
OTU5801	Actinobacteria	*CL500-29_marine_group*	1	50	0.40	2325.41	0.30
OTU10012	Proteobacteria	*Cellvibrio*	1	35	0.38	470.39	0.51
OTU4644	Proteobacteria	*alphaI_cluster*	1	8	0.32	35.45	0.50

Table S6 Topological properties of microbial community related to C3% in Bauyangdian Lake.

Nodes	Phylum	Genus	Modularity	Degree	Closness centrality	Betweenness centrality	Clustering
OTU8159	Proteobacteria	*norank_f__Mitochondria*	4	31	0.36	968.69	0.33
OTU4318	Actinobacteria	*norank_o__PeM15*	3	17	0.33	660.73	0.35
OTU1043	Planctomycetes	*norank_f__Gemmataceae*	2	35	0.35	683.95	0.47
OTU10548	Bacteroidetes	*Flavobacterium*	2	24	0.36	635.82	0.51
OTU1619	Cyanobacteria	*norank_o__Chloroplast*	2	42	0.37	640.22	0.45
OTU1630	Actinobacteria	*CL500-29_marine_group*	2	40	0.37	456.93	0.46
OTU1748	Actinobacteria	*CL500-29_marine_group*	2	54	0.38	781.67	0.42
OTU1988	Actinobacteria	*norank_o__Gaiellales*	2	48	0.38	1053.17	0.40
OTU2246	Bacteroidetes	*Flavobacterium*	2	30	0.38	876.93	0.48
OTU2247	Bacteroidetes	*Flavobacterium*	2	10	0.31	46.96	0.78
OTU2316	Bacteroidetes	*Fluviicola*	2	26	0.36	732.15	0.43
OTU2721	Actinobacteria	*CL500-29_marine_group*	2	59	0.40	1643.05	0.35
OTU3354	Actinobacteria	*Aurantimicrobium*	2	36	0.36	689.99	0.40
OTU3358	Bacteroidetes	*Dinghuibacter*	2	41	0.37	663.75	0.44
OTU3359	Actinobacteria	*unclassified_c__Actinobacteria*	2	44	0.38	799.07	0.39
OTU4279	Planctomycetes	*CL500-3*	2	32	0.37	286.80	0.48
OTU4334	Cyanobacteria	*norank_o__Chloroplast*	2	38	0.38	718.37	0.43
OTU4613	Actinobacteria	*norank_o__PeM15*	2	21	0.34	492.61	0.49
OTU5117	Proteobacteria	*Roseomonas*	2	12	0.33	251.78	0.36
OTU5491	Actinobacteria	*Candidatus_Limnoluna*	2	38	0.37	471.64	0.43
OTU6234	Bacteroidetes	*norank_f__Chitinophagaceae*	2	27	0.36	377.05	0.46
OTU6236	Bacteroidetes	*Flavobacterium*	2	33	0.37	533.55	0.45
OTU7082	Actinobacteria	*hgcI_clade*	2	49	0.37	763.34	0.41
OTU7100	Bacteroidetes	*Flavobacterium*	2	39	0.38	1053.93	0.43
OTU8195	Cyanobacteria	*norank_o__Chloroplast*	2	21	0.34	139.84	0.60
OTU9287	Verrucomicrobia	*Luteolibacter*	2	12	0.32	332.18	0.45

Reference

Chen, Y, Senesi, N, Schnitzer, M, 1977. Information provided on humic substances by E4/E6 ratios 1. Soil Sci. Soc. Am. J. 41:352-358.

Helms, J R, Stubbins, A, Ritchie, J D, Minor, E C, Kieber, D J, Mopper, K, 2008. Absorption spectral slopes and slope ratios as indicators of molecular weight, source, and photobleaching of chromophoric dissolved organic matter. Limnol. Oceanogr. 53:955-969.

Huguet, A, Vacher, L, Relexans, S, Saubusse, S, Froidefond, J-M, Parlanti, E, 2009. Properties of fluorescent dissolved organic matter in the Gironde Estuary. Org Geochem 40:706-719.

Lavonen, E, Kothawala, D, Tranvik, L, Gonsior, M, Schmitt-Kopplin, P, Köhler, S, 2015. Tracking changes in the optical properties and molecular composition of dissolved organic matter during drinking water production. Water Res 85:286-294.

Lawaetz, A J, Stedmon, C A, 2009. Fluorescence intensity calibration using the Raman scatter peak of water. Appl. Spectrosc. 63:936-940.

Li, P, Hur, J, 2017. Utilization of UV-Vis spectroscopy and related data analyses for dissolved organic matter (DOM) studies: A review. Crit. Rev. Env. Sci. Tec. 47:131-154.

Ohno, T, 2002. Fluorescence inner-filtering correction for determining the humification index of dissolved organic matter. Environ. Sci. Technol. 36:742-746.

Shafiquzzaman, M, Ahmed, A T, Azam, M S, Razzak, A, Askri, B, Hassan, H F, Ravikumar, B, Okuda, T, 2014. Identification and characterization of dissolved organic matter sources in Kushiro river impacted by a wetland. Ecol. Eng. 70:459-464.

Stedmon, C A, Bro, R, 2008. Characterizing dissolved organic matter fluorescence with parallel factor analysis: a tutorial. Limnol. Oceanogr-Meth 6:572-579.

Zhang, Y, Liu, M, Qin, B, Feng, S, 2009. Photochemical degradation of chromophoric-dissolved organic matter exposed to simulated UV-B and natural solar radiation. Hydrobiologia 627:159-168.

Zhou, S, Zhang, Y, Huang, T, Liu, Y, Fang, K, Zhang, C, 2019. Microbial aerobic denitrification dominates nitrogen losses from reservoir ecosystem in the spring of Zhoucun reservoir. Sci. Total Environ. 651:998-1010.
